# Optimized mixed Markov models for motif identification

**DOI:** 10.1186/1471-2105-7-279

**Published:** 2006-06-02

**Authors:** Weichun Huang, David M Umbach, Uwe Ohler, Leping Li

**Affiliations:** 1 Bioinformatics Research Center, North Carolina State University, Raleigh, NC 27606, USA; 2 Biostatistics Branch, The National Institute of Environmental Health Sciences, National Institutes of Health, RTP, NC 27709, USA; 3 Institute for Genome Sciences & Policy, Duke University Medical Center, Durham, NC 27708, USA

## Abstract

**Background:**

Identifying functional elements, such as transcriptional factor binding sites, is a fundamental step in reconstructing gene regulatory networks and remains a challenging issue, largely due to limited availability of training samples.

**Results:**

We introduce a novel and flexible model, the Optimized Mixture Markov model (OMiMa), and related methods to allow adjustment of model complexity for different motifs. In comparison with other leading methods, OMiMa can incorporate more than the NNSplice's pairwise dependencies; OMiMa avoids model over-fitting better than the Permuted Variable Length Markov Model (PVLMM); and OMiMa requires smaller training samples than the Maximum Entropy Model (MEM). Testing on both simulated and actual data (regulatory *cis*-elements and splice sites), we found OMiMa's performance superior to the other leading methods in terms of prediction accuracy, required size of training data or computational time. Our OMiMa system, to our knowledge, is the only motif finding tool that incorporates automatic selection of the best model. OMiMa is freely available at [1].

**Conclusion:**

Our optimized mixture of Markov models represents an alternative to the existing methods for modeling dependent structures within a biological motif. Our model is conceptually simple and effective, and can improve prediction accuracy and/or computational speed over other leading methods.

## Background

Biological sequences, including DNA, RNA and proteins, contain functionally important motifs, such as transcription factor binding sites (TFBS), RNA splice sites, and protein domains. With the increasing-availability of genome sequences, identification of such functional motifs not only plays important roles in gene finding and function prediction but also is a fundamental step in reconstructing gene regulatory networks and in revealing gene evolutionary mechanisms [2-6].

A commonly used model for motif identification is the Weight Matrix Model (WMM) proposed by Staden [7], also called the Position Weight Matrix (PWM) or Mononucleotide Weight Matrix (MWM). A PWM is usually generated from a set of aligned instances of known motif sequences, using the observed position-specific base frequencies and/or prior information. Stormo and Fields [8] showed that the PWM score of a motif is proportional to the total binding energy contributed by individual bases. PWM has been used by many motif identification programs, *e.g*., Matlnspector [9] and Match [10], and performs reasonably well for motif identification. While a PWM can capture both nucleotide preferences at each position and different levels of position specificity, it does not account for functional dependencies between positions. Recent studies [11-15] indicate that there are often important interactions between positions, adjacent as well as non-adjacent, within a motif. The inability of the PWM to capture such dependencies is a limitation as the PWM model often produces a large number of false positives in a genome-wide scan [16].

Many models have been developed to incorporate position dependencies. Motif models, such as the Dinucleotide Weight Matrix Model (DWMM) [17] and the Weight Array Model (WAM) [18], can incorporate dependencies between adjacent positions. To incorporate further dependencies of non-adjacent positions, Ponomarenko *et al. *[19] extended DWMM by introducing the Oligonucleotide Weight Matrix model, which includes a comprehensive set of oliogonucleotide matrices classified into 5 biological function categories. A WAM could also be extended to a high order WAM in principle, *e.g*., windowed *2*^nd ^order WAM [2]. However, the exponentially increased number of parameters of these models makes them impractical due to insufficient training data. To address the weaknesses of WAM in incorporating long-range interactions, Burge and Karlin [2] proposed the Maximal Dependence Decomposition (MDD) model, which has a binary tree structure formed by a set of conditional WAMs. While the MDD model can capture non-adjacent dependencies through the conditional WAM models, it still requires a rather large number of training sequences, which are partitioned into smaller subsets to train all conditional WAMs. To alleviate the requirement of a large training set, Cai *et al. *[20] developed a Bayesian tree to model dependencies within RNA splice sites; Ellrott *et al. *[21] suggested a position order optimized Markov chain model, which reorders motif positions to bring distant but dependent positions into near neighbors. More recently, several other models have been developed, including Bayesian networks for modeling protein-DNA binding sites [22], Maximum Entropy Model (MEM) for splice site identification [23], Permuted Variable Length Markov Model (PVLMM) for finding transcription factor binding sites and splice sites [24]. For a biological motif with position dependencies, these models can show improvement in prediction accuracy over the models that assume independence. Incorporating position dependencies can also improve the accuracy of *de novo *motif discovery [25].

In this paper, we present a new and flexible motif model, the OMiMa, to incorporate position dependencies within a motif. OMiMa can not only adjust model complexity according to motif dependency structures but also minimize model complexity without compromising prediction accuracy. As an integrated part of OMiMa, we also introduce the Directed Neighbor-Joining (DNJ) method to optimally rearrange positions to minimize Markov order. We then describe and discuss the methods for selecting the best model. We implement our model into the OMiMa system that is freely available to the public.

## Results

### Mixed Markov models

Let *X*_*i *_be the discrete random variable associated with position *i *in a biological motif **X **of length *w*. For DNA sequences, *X*_*i *_takes values from set B = {*A, C, G, T*}; and for protein sequences, *X*_*i *_takes values from 20 different amino acids. *X*_*i *_follows a multinomial distribution. Let Xik
 MathType@MTEF@5@5@+=feaafiart1ev1aaatCvAUfKttLearuWrP9MDH5MBPbIqV92AaeXatLxBI9gBaebbnrfifHhDYfgasaacH8akY=wiFfYdH8Gipec8Eeeu0xXdbba9frFj0=OqFfea0dXdd9vqai=hGuQ8kuc9pgc9s8qqaq=dirpe0xb9q8qiLsFr0=vr0=vr0dc8meaabaqaciaacaGaaeqabaqabeGadaaakeaaieqacqWFybawdaqhaaWcbaGaemyAaKgabaGaem4AaSgaaaaa@30D2@ = *X*_*i*-*k*_...*X*_*i*-1 _and xik
 MathType@MTEF@5@5@+=feaafiart1ev1aaatCvAUfKttLearuWrP9MDH5MBPbIqV92AaeXatLxBI9gBaebbnrfifHhDYfgasaacH8akY=wiFfYdH8Gipec8Eeeu0xXdbba9frFj0=OqFfea0dXdd9vqai=hGuQ8kuc9pgc9s8qqaq=dirpe0xb9q8qiLsFr0=vr0=vr0dc8meaabaqaciaacaGaaeqabaqabeGadaaakeaaieqacqWF4baEdaqhaaWcbaGaemyAaKgabaGaem4AaSgaaaaa@3112@ = *x*_*i*-*k*_...*x*_*i*-1_, where *k *= 0,..., *w *- 1; upper case **X **(*X*_*i*_) is a random variable and lower case **x** (*x*_*i*_) is a particular value. The xi0
 MathType@MTEF@5@5@+=feaafiart1ev1aaatCvAUfKttLearuWrP9MDH5MBPbIqV92AaeXatLxBI9gBaebbnrfifHhDYfgasaacH8akY=wiFfYdH8Gipec8Eeeu0xXdbba9frFj0=OqFfea0dXdd9vqai=hGuQ8kuc9pgc9s8qqaq=dirpe0xb9q8qiLsFr0=vr0=vr0dc8meaabaqaciaacaGaaeqabaqabeGadaaakeaacqWG4baEdaqhaaWcbaGaemyAaKgabaGaeGimaadaaaaa@309B@ denotes an empty sequence and Pr(Xi0
 MathType@MTEF@5@5@+=feaafiart1ev1aaatCvAUfKttLearuWrP9MDH5MBPbIqV92AaeXatLxBI9gBaebbnrfifHhDYfgasaacH8akY=wiFfYdH8Gipec8Eeeu0xXdbba9frFj0=OqFfea0dXdd9vqai=hGuQ8kuc9pgc9s8qqaq=dirpe0xb9q8qiLsFr0=vr0=vr0dc8meaabaqaciaacaGaaeqabaqabeGadaaakeaaieqacqWFybawdaqhaaWcbaGaemyAaKgabaGaeGimaadaaaaa@3061@ = xi0
 MathType@MTEF@5@5@+=feaafiart1ev1aaatCvAUfKttLearuWrP9MDH5MBPbIqV92AaeXatLxBI9gBaebbnrfifHhDYfgasaacH8akY=wiFfYdH8Gipec8Eeeu0xXdbba9frFj0=OqFfea0dXdd9vqai=hGuQ8kuc9pgc9s8qqaq=dirpe0xb9q8qiLsFr0=vr0=vr0dc8meaabaqaciaacaGaaeqabaqabeGadaaakeaacqWG4baEdaqhaaWcbaGaemyAaKgabaGaeGimaadaaaaa@309B@) = 1. Additionally, let *X*_-*j *_= *X*_*w*-*j*_, *x*_-*j *_= *x*_*w*-*j*_, where *j *= 0...*w *- 1. If one uses the *k*^*th *^order Markov model (*M*_*k*_), the probability of observing a motif sequence x is just the product of conditional/transition probabilities. Let MkL
 MathType@MTEF@5@5@+=feaafiart1ev1aaatCvAUfKttLearuWrP9MDH5MBPbIqV92AaeXatLxBI9gBaebbnrfifHhDYfgasaacH8akY=wiFfYdH8Gipec8Eeeu0xXdbba9frFj0=OqFfea0dXdd9vqai=hGuQ8kuc9pgc9s8qqaq=dirpe0xb9q8qiLsFr0=vr0=vr0dc8meaabaqaciaacaGaaeqabaqabeGadaaakeaacqWGnbqtdaqhaaWcbaGaem4AaSgabaGaemitaWeaaaaa@307C@ be a *k*^*th *^order Markov model of a linear chain, and MkC
 MathType@MTEF@5@5@+=feaafiart1ev1aaatCvAUfKttLearuWrP9MDH5MBPbIqV92AaeXatLxBI9gBaebbnrfifHhDYfgasaacH8akY=wiFfYdH8Gipec8Eeeu0xXdbba9frFj0=OqFfea0dXdd9vqai=hGuQ8kuc9pgc9s8qqaq=dirpe0xb9q8qiLsFr0=vr0=vr0dc8meaabaqaciaacaGaaeqabaqabeGadaaakeaacqWGnbqtdaqhaaWcbaGaem4AaSgabaGaem4qameaaaaa@306A@ be a *k*^*th *^order Markov model of a circular chain. The probability of a motif sequence is given by equation (1) for a linear chain and equation (2) for a circular chain, respectively.

Pr⁡(x|MkL)=Pr⁡(Xk+1k=xk+1k)∏i=k+1wPr⁡(Xi=xi|Xik=xik)     (1)
 MathType@MTEF@5@5@+=feaafiart1ev1aaatCvAUfKttLearuWrP9MDH5MBPbIqV92AaeXatLxBI9gBaebbnrfifHhDYfgasaacH8akY=wiFfYdH8Gipec8Eeeu0xXdbba9frFj0=OqFfea0dXdd9vqai=hGuQ8kuc9pgc9s8qqaq=dirpe0xb9q8qiLsFr0=vr0=vr0dc8meaabaqaciaacaGaaeqabaqabeGadaaakeaacyGGqbaucqGGYbGCcqGGOaakieqacqWF4baEcqGG8baFcqWGnbqtdaqhaaWcbaGaem4AaSgabaGaemitaWeaaOGaeiykaKIaeyypa0JagiiuaaLaeiOCaiNaeiikaGIae8hwaG1aa0baaSqaaiabdUgaRjabgUcaRiabigdaXaqaaiabdUgaRbaakiabg2da9iab=Hha4naaDaaaleaacqWGRbWAcqGHRaWkcqaIXaqmaeaacqWGRbWAaaGccqGGPaqkdaqeWbqaaiGbccfaqjabckhaYjabcIcaOiabdIfaynaaBaaaleaacqWGPbqAaeqaaOGaeyypa0JaemiEaG3aaSbaaSqaaiabdMgaPbqabaGccqGG8baFcqWFybawdaqhaaWcbaGaemyAaKgabaGaem4AaSgaaOGaeyypa0Jae8hEaG3aa0baaSqaaiabdMgaPbqaaiabdUgaRbaakiabcMcaPaWcbaGaemyAaKMaeyypa0Jaem4AaSMaey4kaSIaeGymaedabaGaem4DaChaniabg+GivdGccaWLjaGaaCzcamaabmaabaGaeGymaedacaGLOaGaayzkaaaaaa@6D8E@

Pr⁡(x|MkC)=∏i=1wPr⁡(Xi=xi|Xik=xik)     (2)
 MathType@MTEF@5@5@+=feaafiart1ev1aaatCvAUfKttLearuWrP9MDH5MBPbIqV92AaeXatLxBI9gBaebbnrfifHhDYfgasaacH8akY=wiFfYdH8Gipec8Eeeu0xXdbba9frFj0=OqFfea0dXdd9vqai=hGuQ8kuc9pgc9s8qqaq=dirpe0xb9q8qiLsFr0=vr0=vr0dc8meaabaqaciaacaGaaeqabaqabeGadaaakeaacyGGqbaucqGGYbGCcqGGOaakieqacqWF4baEcqGG8baFcqWGnbqtdaqhaaWcbaGaem4AaSgabaGaem4qameaaOGaeiykaKIaeyypa0ZaaebCaeaacyGGqbaucqGGYbGCcqGGOaakcqWGybawdaWgaaWcbaGaemyAaKgabeaakiabg2da9iabdIha4naaBaaaleaacqWGPbqAaeqaaOGaeiiFaWNae8hwaG1aa0baaSqaaiabdMgaPbqaaiabdUgaRbaakiabg2da9iab=Hha4naaDaaaleaacqWGPbqAaeaacqWGRbWAaaGccqGGPaqkaSqaaiabdMgaPjabg2da9iabigdaXaqaaiabdEha3bqdcqGHpis1aOGaaCzcaiaaxMaadaqadaqaaiabikdaYaGaayjkaiaawMcaaaaa@59B7@

Compared to a linear Markov chain, a circular Markov chain incorporates additional dependencies that may contain subtle signals that allow the model to distinguish true motifs from false ones, especially when false motifs are similar to true motifs.

Suppose a motif **X **can be divided into *m *independent sub-motifs, that is **X **= **Y**_1_,... **Y**_*m *_and each sub-motif is modeled as an independent Markov chain, that is MX=MY1,…,MYm
 MathType@MTEF@5@5@+=feaafiart1ev1aaatCvAUfKttLearuWrP9MDH5MBPbIqV92AaeXatLxBI9gBaebbnrfifHhDYfgasaacH8akY=wiFfYdH8Gipec8Eeeu0xXdbba9frFj0=OqFfea0dXdd9vqai=hGuQ8kuc9pgc9s8qqaq=dirpe0xb9q8qiLsFr0=vr0=vr0dc8meaabaqaciaacaGaaeqabaqabeGadaaakeaacqWGnbqtdaWgaaWcbaGaemiwaGfabeaakiabg2da9iabd2eannaaBaaaleaacqWGzbqwdaWgaaadbaGaeGymaedabeaaaSqabaGccqGGSaalcqWIMaYscqGGSaalcqWGnbqtdaWgaaWcbaGaemywaK1aaSbaaWqaaiabd2gaTbqabaaaleqaaaaa@3B07@, then the probability of the sequence (x) given the Markov models is:

Pr⁡(X=x|MX)=∏i=1mPr⁡(Yi=yi|MYi)
 MathType@MTEF@5@5@+=feaafiart1ev1aaatCvAUfKttLearuWrP9MDH5MBPbIqV92AaeXatLxBI9gBaebbnrfifHhDYfgasaacH8akY=wiFfYdH8Gipec8Eeeu0xXdbba9frFj0=OqFfea0dXdd9vqai=hGuQ8kuc9pgc9s8qqaq=dirpe0xb9q8qiLsFr0=vr0=vr0dc8meaabaqaciaacaGaaeqabaqabeGadaaakeaacyGGqbaucqGGYbGCcqGGOaakieqacqWFybawcqGH9aqpcqWF4baEcqGG8baFcqWGnbqtdaWgaaWcbaGaemiwaGfabeaakiabcMcaPiabg2da9maarahabaGagiiuaaLaeiOCaiNaeiikaGIae8xwaK1aaSbaaSqaaiabdMgaPbqabaGccqGH9aqpcqWF5bqEdaWgaaWcbaGaemyAaKgabeaakiabcYha8jabd2eannaaBaaaleaacqWGzbqwdaWgaaadbaGaemyAaKgabeaaaSqabaGccqGGPaqkaSqaaiabdMgaPjabg2da9iabigdaXaqaaiabd2gaTbqdcqGHpis1aaaa@5170@

**Figure 1:**
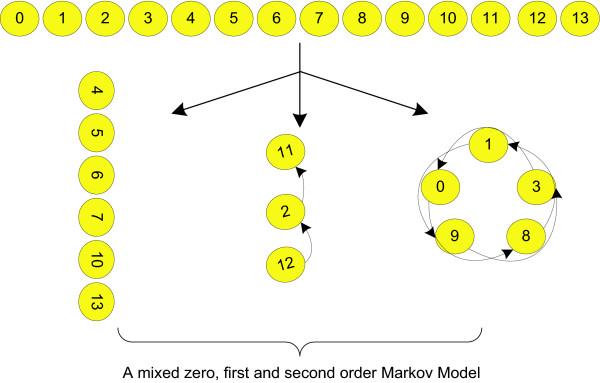
**The graphic representation of a mixture of Markov models**. A graphic representation of a mixture of Markov models. On the top is a motif of length 14 bases. On the left, 6 positions, which are independent of each other and all other positions, form a 0^*th *^order Markov chain. In the middle, 3 positions form a linear chain of 1^*st *^order Markov model. On the right, the remaining positions that closely depend on each other form a circular chain of the 2^*nd *^order Markov model.

**Figure 2:**
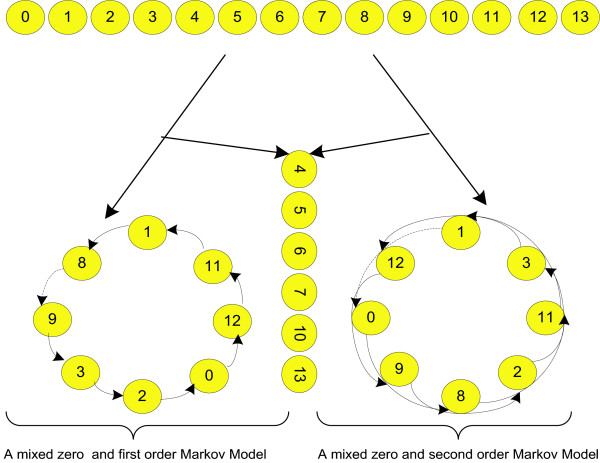
**The graphic representation of the 0-k mixture model for TFBS**. The simple mixture of Markov models for TFBS. Since TFBS are short (5–16 bases), a mixture model consisting of the 0^*th *^order and 1^*st*^/2^*nd *^order Markov chains is generally adequate for predicting new binding sites. The sub-motif formed by independent positions is modeled by a 0^*th *^order Markov order model. The sub-motif forming by the remaining positions is modeled by either a 1^*st *^or 2^*nd *^order Markov chain, which can be either linear (break at dotted arrows) or circular.

These independent Markov models, each of which is position-optimized for its corresponding sub-motif, form an Optimized Mixture of Markov models (OMiMa). An example of OMiMa is illustrated in Figure 1. However, for short motifs such as transcription factor binding sites, we can use a simple mixture of Markov models consisting of only one 0^*th *^order and one *k*^*th *^order chain (Figure 2). For convenience, we refer such a mixture model as *'a 0-k mixture model'*. Since the *k*^*th *^order Markov chain of *'a 0-k mixture model' *can be either linear or circular, we also use terms *'a 0-k mixture linear model' *and *'a 0-k mixture circular model' *to distinguish them. In the following, we describe methods for the general mixture Markov model, while we use the simple 0-k mixture model for our testing.

Conceivably, the different parts of a motif could have distinct roles in the interaction with their partners. Motif positions involved in the same role can be highly dependent, whereas those involved unrelated roles are likely independent. A mixture of Markov models seems an ideal fit by modeling different signals with different sub-models. A 0^*th *^order Markov chain can effectively model strong signals such as those embedded in highly conserved positions where the probability of a certain base occurring is almost one. In addition, positions where base composition contributes little or nothing to motif function need no more complex model than a 0^*th *^order Markov model. On the other hand, a higher order Markov model is necessary for detecting subtle dependency signals that can be essential for distinguishing true motifs from false ones.

### Motif dissection

To apply the mixture of Markov models to a motif, the first step is to dissect the motif into several independent sub-motifs, each of which is modeled as a Markov chain. For a given set of sequences of a motif, we employ chi-square tests to find significant pairwise dependencies between positions within the motif (see also [21]). Based on pairwise dependencies, motif positions are grouped into independent sets, each forming a Markov chain. The outline of our procedure for grouping motif positions is described in the following steps.

1. Calculate base frequencies for each position, and find highly conserved positions where the observed frequency of a certain base (almost) equals 1. These conserved positions then are put into set *H *as defined below.

H={i:max⁡x∈Bf(i,x)≈1}
 MathType@MTEF@5@5@+=feaafiart1ev1aaatCvAUfKttLearuWrP9MDH5MBPbIqV92AaeXatLxBI9gBaebbnrfifHhDYfgasaacH8akY=wiFfYdH8Gipec8Eeeu0xXdbba9frFj0=OqFfea0dXdd9vqai=hGuQ8kuc9pgc9s8qqaq=dirpe0xb9q8qiLsFr0=vr0=vr0dc8meaabaqaciaacaGaaeqabaqabeGadaaakeaacqWGibascqGH9aqpcqGG7bWEcqWGPbqAcqGG6aGodaWfqaqaaiGbc2gaTjabcggaHjabcIha4bWcbaGaemiEaGNaeyicI4SaeeOqaieabeaakiabdAgaMjabcIcaOiabdMgaPjabcYcaSiabdIha4jabcMcaPiabgIKi7kabigdaXiabc2ha9baa@45EF@

where *f*(*i, x*) is the frequency of base x at position i, and B is the set of bases.

2. Place remaining positions in the set *M*, and calculate pairwise chi-square values for every pair of positions in *M*.

χi,j2=∑xi∈Bi∑xj∈Bj(O(xi,xj)−E(xi,xj))2E(xi,xj)     (3)
 MathType@MTEF@5@5@+=feaafiart1ev1aaatCvAUfKttLearuWrP9MDH5MBPbIqV92AaeXatLxBI9gBaebbnrfifHhDYfgasaacH8akY=wiFfYdH8Gipec8Eeeu0xXdbba9frFj0=OqFfea0dXdd9vqai=hGuQ8kuc9pgc9s8qqaq=dirpe0xb9q8qiLsFr0=vr0=vr0dc8meaabaqaciaacaGaaeqabaqabeGadaaakeaaiiGacqWFhpWydaqhaaWcbaGaemyAaKMaeiilaWIaemOAaOgabaGaeGOmaidaaOGaeyypa0ZaaabuaeaadaaeqbqaamaalaaabaGaeiikaGIaem4ta8KaeiikaGIaemiEaG3aaSbaaSqaaiabdMgaPbqabaGccqGGSaalcqWG4baEdaWgaaWcbaGaemOAaOgabeaakiabcMcaPiabgkHiTiabdweafjabcIcaOiabdIha4naaBaaaleaacqWGPbqAaeqaaOGaeiilaWIaemiEaG3aaSbaaSqaaiabdQgaQbqabaGccqGGPaqkcqGGPaqkdaahaaWcbeqaaiabikdaYaaaaOqaaiabdweafjabcIcaOiabdIha4naaBaaaleaacqWGPbqAaeqaaOGaeiilaWIaemiEaG3aaSbaaSqaaiabdQgaQbqabaGccqGGPaqkaaaaleaacqWG4baEdaWgaaadbaGaemOAaOgabeaaliabgIGiolabbkeacnaaBaaameaacqWGQbGAaeqaaaWcbeqdcqGHris5aaWcbaGaemiEaG3aaSbaaWqaaiabdMgaPbqabaWccqGHiiIZcqqGcbGqdaWgaaadbaGaemyAaKgabeaaaSqab0GaeyyeIuoakiaaxMaacaWLjaWaaeWaaeaacqaIZaWmaiaawIcacaGLPaaaaaa@6BBF@

where B_*i *_and B_*j *_are the sets of bases observed in positions *i *and *j*, respectively; *O*(*x*_*i*_, *x*_*j*_) and *E*(*x*_*i*_, *x*_*j*_) are the observed and expected counts of pair (*x*_*i*_, *x*_*j*_), respectively. *E*(*x*_*i*_, *x*_*j*_) is the product of observed base frequencies *x*_*i *_and *x*_*j*_. The degrees of freedom of this test is (|B_*i*_| - 1) × (|B_*j*_| - 1), where |B_*i*_| and |B_*j*_| are the number of different bases in sets B_*i *_and B_*j*_, respectively.

3. Based on the above *χ*^2 ^tests, find all positions that show little dependence on any other positions in *M*, and move them to the set *I*, as defined by

I={i=min⁡i≠j;i,j∈Mpi,j>α}
 MathType@MTEF@5@5@+=feaafiart1ev1aaatCvAUfKttLearuWrP9MDH5MBPbIqV92AaeXatLxBI9gBaebbnrfifHhDYfgasaacH8akY=wiFfYdH8Gipec8Eeeu0xXdbba9frFj0=OqFfea0dXdd9vqai=hGuQ8kuc9pgc9s8qqaq=dirpe0xb9q8qiLsFr0=vr0=vr0dc8meaabaqaciaacaGaaeqabaqabeGadaaakeaacqWGjbqscqGH9aqpdaGadaqaaiabdMgaPjabg2da9maaxababaGagiyBa0MaeiyAaKMaeiOBa4galeaacqWGPbqAcqGHGjsUcqWGQbGAcqGG7aWocqWGPbqAcqGGSaalcqWGQbGAcqGHiiIZcqWGnbqtaeqaaOGaemiCaa3aaSbaaSqaaiabdMgaPjabcYcaSiabdQgaQbqabaGccqGH+aGpiiGacqWFXoqyaiaawUhacaGL9baaaaa@4B65@

Here *p*_*i,j *_is the p-value corresponding to χi,j2
 MathType@MTEF@5@5@+=feaafiart1ev1aaatCvAUfKttLearuWrP9MDH5MBPbIqV92AaeXatLxBI9gBaebbnrfifHhDYfgasaacH8akY=wiFfYdH8Gipec8Eeeu0xXdbba9frFj0=OqFfea0dXdd9vqai=hGuQ8kuc9pgc9s8qqaq=dirpe0xb9q8qiLsFr0=vr0=vr0dc8meaabaqaciaacaGaaeqabaqabeGadaaakeaaiiGacqWFhpWydaqhaaWcbaGaemyAaKMaeiilaWIaemOAaOgabaGaeGOmaidaaaaa@3321@, and *α *is the significance level, *e.g*., 0.05.

4. The remaining positions in *M *are further grouped into subsets by iterating the following rules:

(a) Set s = 1.

(b) Calculate *θ*_*i *_= ∑_*j*∈*M*,*j*≠*i *_*δ*(*p*_*i,j *_<*α*) for each position *i *in *M*, where *δ *is a 0/1 indicator function. Find the largest *θ*_*i*_, and move position *i *and all positions *j *that *p*_*i,j *_<*α *from *M *into a new set *C*_*s*_.

(c) For each remaining position, check if it significantly depends on any position in *C*_*s*_. If it does, then move it from *M *into *C*_*s*_.

(d) If *M *is not empty, update s = s+1 and go back to step (b).

Step 4 above essentially groups positions into independent subsets, each potentially forming a functional unit. For the special 0-k mixture model, we simply set *M *= *C*_1 _at this step.

### Markov chain optimization

The next step is to arrange the positions in each subset into a Markov chain. Since the positions in sets *H *and *I *are independent of each other, they can be arranged in their natural order to form a 0^*th *^order Markov chain. The positions in *H *can also be treated differently from those in set *I *in motif identification by requiring a perfect match for a true site. Sets *C*_*s *_are different. The position arrangement for each set *C*_*s *_needs to be optimized so that the Markov model can account for most dependencies while minimizing the Markov order. For a given set *C*_*s*_, we use the median (*K*_*s*_) of *θ *(*θ *= {*θ*_*j*_, *j *∈ *C*_*s*_}) as the maximum order of its potential Markov model. We then optimize position arrangement for the *k*^*th *^order Markov chain (*k *= 0,..., *K*_*s*_) by the Directed Neighbor-Joining (DNJ) method described below.

**Figure 3:**
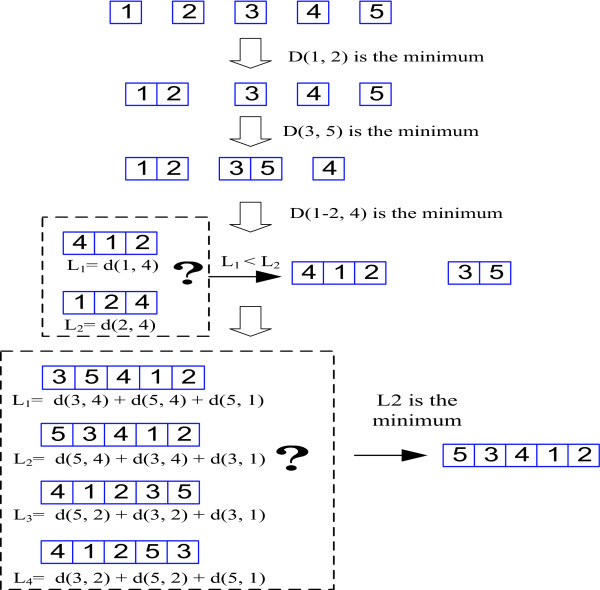
**Illustration of the DNJ method for Markov chain optimization**. An example of the DNJ method to optimize the 2^*nd *^order Markov chain.

The neighbor-joining (NJ) method proposed by Saitou and Nei [26] is a well-known distance method for phylogenetic tree reconstruction. The principle of the NJ method is to find pairs of operational taxonomic units that minimize the total branch length at each stage of clustering. Our DNJ method is based on the exactly same principle. The only major difference is that DNJ needs to consider the direction in joining two nearest neighbors to form a new node while NJ does not. Instead of producing a phylogenetic tree as the NJ method does, DNJ method creates a chain structure, which arranges closely dependent positions as the nearest neighbors. The DNJ method for constructing a *k*^*th *^order Markov chain from a given subset (*C*_*s*_) of motif positions is described in the following steps (see Figure 3 for an example).

1. For a given set *C*_*s*_, put each position in the set into a different vector. Here a vector is represented by a letter, an arrow at the top of the letter may be used to indicate the direction of a vector, *e.g., a *stands for either a→
 MathType@MTEF@5@5@+=feaafiart1ev1aaatCvAUfKttLearuWrP9MDH5MBPbIqV92AaeXatLxBI9gBaebbnrfifHhDYfgasaacH8akY=wiFfYdH8Gipec8Eeeu0xXdbba9frFj0=OqFfea0dXdd9vqai=hGuQ8kuc9pgc9s8qqaq=dirpe0xb9q8qiLsFr0=vr0=vr0dc8meaabaqaciaacaGaaeqabaqabeGadaaakeaadaWhcaqaaiabdggaHbGaay51Gaaaaa@2FAB@ or a←
 MathType@MTEF@5@5@+=feaafiart1ev1aaatCvAUfKttLearuWrP9MDH5MBPbIqV92AaeXatLxBI9gBaebbnrfifHhDYfgasaacH8akY=wiFfYdH8Gipec8Eeeu0xXdbba9frFj0=OqFfea0dXdd9vqai=hGuQ8kuc9pgc9s8qqaq=dirpe0xb9q8qiLsFr0=vr0=vr0dc8meaabaqaciaacaGaaeqabaqabeGadaaakeaadaWhbaqaaiabdggaHbGaayP1Gaaaaa@2FA9@. If a→
 MathType@MTEF@5@5@+=feaafiart1ev1aaatCvAUfKttLearuWrP9MDH5MBPbIqV92AaeXatLxBI9gBaebbnrfifHhDYfgasaacH8akY=wiFfYdH8Gipec8Eeeu0xXdbba9frFj0=OqFfea0dXdd9vqai=hGuQ8kuc9pgc9s8qqaq=dirpe0xb9q8qiLsFr0=vr0=vr0dc8meaabaqaciaacaGaaeqabaqabeGadaaakeaadaWhcaqaaiabdggaHbGaay51Gaaaaa@2FAB@ = (1, 2, 3), then a←
 MathType@MTEF@5@5@+=feaafiart1ev1aaatCvAUfKttLearuWrP9MDH5MBPbIqV92AaeXatLxBI9gBaebbnrfifHhDYfgasaacH8akY=wiFfYdH8Gipec8Eeeu0xXdbba9frFj0=OqFfea0dXdd9vqai=hGuQ8kuc9pgc9s8qqaq=dirpe0xb9q8qiLsFr0=vr0=vr0dc8meaabaqaciaacaGaaeqabaqabeGadaaakeaadaWhbaqaaiabdggaHbGaayP1Gaaaaa@2FA9@ = (3, 2, 1), a→
 MathType@MTEF@5@5@+=feaafiart1ev1aaatCvAUfKttLearuWrP9MDH5MBPbIqV92AaeXatLxBI9gBaebbnrfifHhDYfgasaacH8akY=wiFfYdH8Gipec8Eeeu0xXdbba9frFj0=OqFfea0dXdd9vqai=hGuQ8kuc9pgc9s8qqaq=dirpe0xb9q8qiLsFr0=vr0=vr0dc8meaabaqaciaacaGaaeqabaqabeGadaaakeaadaWhcaqaaiabdggaHbGaay51Gaaaaa@2FAB@a←
 MathType@MTEF@5@5@+=feaafiart1ev1aaatCvAUfKttLearuWrP9MDH5MBPbIqV92AaeXatLxBI9gBaebbnrfifHhDYfgasaacH8akY=wiFfYdH8Gipec8Eeeu0xXdbba9frFj0=OqFfea0dXdd9vqai=hGuQ8kuc9pgc9s8qqaq=dirpe0xb9q8qiLsFr0=vr0=vr0dc8meaabaqaciaacaGaaeqabaqabeGadaaakeaadaWhbaqaaiabdggaHbGaayP1Gaaaaa@2FA9@ = (1, 2, 3, 3, 2, 1), and a→
 MathType@MTEF@5@5@+=feaafiart1ev1aaatCvAUfKttLearuWrP9MDH5MBPbIqV92AaeXatLxBI9gBaebbnrfifHhDYfgasaacH8akY=wiFfYdH8Gipec8Eeeu0xXdbba9frFj0=OqFfea0dXdd9vqai=hGuQ8kuc9pgc9s8qqaq=dirpe0xb9q8qiLsFr0=vr0=vr0dc8meaabaqaciaacaGaaeqabaqabeGadaaakeaadaWhcaqaaiabdggaHbGaay51Gaaaaa@2FAB@a→
 MathType@MTEF@5@5@+=feaafiart1ev1aaatCvAUfKttLearuWrP9MDH5MBPbIqV92AaeXatLxBI9gBaebbnrfifHhDYfgasaacH8akY=wiFfYdH8Gipec8Eeeu0xXdbba9frFj0=OqFfea0dXdd9vqai=hGuQ8kuc9pgc9s8qqaq=dirpe0xb9q8qiLsFr0=vr0=vr0dc8meaabaqaciaacaGaaeqabaqabeGadaaakeaadaWhcaqaaiabdggaHbGaay51Gaaaaa@2FAB@ = (1, 2, 3, 1, 2, 3). Initially, each vector has only one position.

2. Create an initial distance matrix (*d*) whose elements are *d*(*u, v*) = *p*_*i,j*_, where *i *is the position in vector *u, j *is the position in vector *v*, and *p*_*i,j *_is the p-value of chi-square test described above.

3. Convert the distance matrix *d *to the transformed distance matrix *D*, whose elements are *D*(*u, v*), by the following conversion function (see [26]):



Where *V *is the number of vectors under consideration, and its value decreases by 1 in each iteration.

4. Find the minimum *D*(*u, v*) in *D*. Then a new vector *x *is formed by joining vector *u *and *v *according to **Algorithm 1 **[see Additional file 1] for a *k*^*th *^order Markov chain.

5. Update the matrix *d *by replacing *u *and *v *by *x*. The distance of *x *to other remaining vector *y *is defined by:

*d*(*x, y*) = (*d*(*u, y*) + *d*(*v, y*) - *d*(*u, v*))/2

6. Go back to step 3 if the number of vectors in *C*_*s *_is larger than 2, otherwise join the last two vectors according to **Algorithm 1**.

The order of positions in the final vector is the optimized linear chain for Markov model. Joining the first position to the last position in the vector forms a circular chain. A linear chain could be further optimized by forming a circular chain first from the final vector, then breaking the circular chain between positions with the weakest dependency, *e.g*., between positions *i *and *j *where *p*_*i,j *_is the largest or the log-likelihood of the corresponding linear chain model is maximized. DNJ not only optimizes position order for linear chain models but also improves circular chain models, particularly when the order of Markov model is low, *e.g*., 1^*st *^or 2^*nd *^order Markov models.

### Model selection

Many different mixtures of Markov models can be formed from the combination of different Markov chains. It is essential to choose the model that minimizes prediction error. In model selection, we first fit each model using maximum likelihood smoothed by a Dirichlet prior [see Additional file 1], then compute either the Akaike information criterion (AIC) [27] or the Bayesian information criterion (BIC) [28]. The model with the minimum value of AIC or BIC is selected as the potential best model. Minimizing AIC is the same as choosing the model with the minimum prediction error or loss, while minimizing BIC is equivalent to choosing the model with the largest posterior probability. Nonetheless, AIC and BIC have a similar form:

-2·*loglik *+ *λ*·*DF*

where *λ *= 2 for AIC and *λ *= log(*N*) for BIC (*N *is the number of sequences); *loglik *is the maximized log likelihood of data given the model; DF is the degrees of freedom (number of free parameters). We replace DF with the effective degrees of freedom (EDF) in calculating AIC or BIC of the mixture of Markov models, which enables an appropriate model to be selected (see sub-section *Effective degrees of freedom*). There is no clear better choice between AIC and BIC for model selection. AIC tends to choose a model too complex as *N *→ ∞, while BIC tends to choose a model too simple when *N *is small. In our test on 61 different TFBS datasets, whose sample sizes range from 20 to 130, we preferred AIC to BIC for picking an appropriate model.

#### Effective degrees of freedom

Let B be the set of bases (|B| denotes the number of different bases in B), *e.g*., for DNA sequences B = {*A, C, G, T*} (|B| = 4). For a motif of length *w*, the DF for a *k *order Markov model is (|B|^*k *^- 1) × (*w *- *k*) for a linear Markov chain; and (|B|^*k *^- 1) × *w *for a circular chain model. That is, the DF increases exponentially as the order of Markov chain increases. As a result, AIC or BIC often pick a simpler mixture model than the best model, especially when |B| is large. Tested on 61 human regulatory motifs from the Transfac database (ver. 7.4) [29], we found that both AIC and BIC selected the 0^*th *^order Markov models for all 61 DNA regulatory motifs when using the DF. To avoid picking overly simple models, we used the EDF described below to calculate AIC and BIC.

Generally, only a subset of bases from B appears in a particular position of a set of biological motifs. The more conserved a position, the fewer bases are in the subset. The EDF for a model is related to the observed bases in training samples. For example, suppose that one would like to estimate nucleotide frequencies occurring in a position in a set of DNA training motifs. If only base A is observed in the position, then one needs to estimate only the frequency of A, the remaining parameters, *i.e*., the frequencies of C, G, T, can be derived from any prior information. Therefore, the actual DF is one in this case. For our mixture of Markov models, the EDF is defined as the number of parameters that are direct estimates of the observed bases in a training motif set. Let *b*_*i *_be the base set observed in a position *i *of a training set of motifs. Additionally, let *h*^*k *^be the sequence of motif positions in the *k*^*th *^order Markov chain, hik
 MathType@MTEF@5@5@+=feaafiart1ev1aaatCvAUfKttLearuWrP9MDH5MBPbIqV92AaeXatLxBI9gBaebbnrfifHhDYfgasaacH8akY=wiFfYdH8Gipec8Eeeu0xXdbba9frFj0=OqFfea0dXdd9vqai=hGuQ8kuc9pgc9s8qqaq=dirpe0xb9q8qiLsFr0=vr0=vr0dc8meaabaqaciaacaGaaeqabaqabeGadaaakeaacqWGObaAdaqhaaWcbaGaemyAaKgabaGaem4AaSgaaaaa@30EC@ be the motif position in the *i*^*th *^element of *h*^*k*^, and ∑|*h*^*k*^| = *w *( |*h*^*k*^| is the number of positions in *h*^*k *^), then we define the EDF for the *k*^*th *^Markov chain as

EDFkL=∑i=k+1|hk|max⁡(bhi−kk×⋯×bhik−1,1)EDFkC=∑i=1|hk|max⁡(bhi−kk×⋯×bhik−1,1)
 MathType@MTEF@5@5@+=feaafiart1ev1aaatCvAUfKttLearuWrP9MDH5MBPbIqV92AaeXatLxBI9gBaebbnrfifHhDYfgasaacH8akY=wiFfYdH8Gipec8Eeeu0xXdbba9frFj0=OqFfea0dXdd9vqai=hGuQ8kuc9pgc9s8qqaq=dirpe0xb9q8qiLsFr0=vr0=vr0dc8meaabaqaciaacaGaaeqabaqabeGadaaakqaabeqaaiabbweafjabbseaejabbAeagnaaBaaaleaacqqGRbWAdaWgaaadbaGaeeitaWeabeaaaSqabaGccqGH9aqpdaaeWaqaaiGbc2gaTjabcggaHjabcIha4jabcIcaOiabdkgaInaaBaaaleaacqWGObaAdaqhaaadbaGaemyAaKMaeyOeI0Iaem4AaSgabaGaem4AaSgaaaWcbeaakiabgEna0kabl+UimjabgEna0kabdkgaInaaBaaaleaacqWGObaAdaqhaaadbaGaemyAaKgabaGaem4AaSgaaaWcbeaakiabgkHiTiabigdaXiabcYcaSiabigdaXiabcMcaPaWcbaGaemyAaKMaeyypa0Jaem4AaSMaey4kaSIaeGymaedabaGaeiiFaWNaemiAaG2aaWbaaWqabeaacqWGRbWAaaWccqGG8baFa0GaeyyeIuoaaOqaaiabbweafjabbseaejabbAeagnaaBaaaleaacqqGRbWAdaWgaaadbaGaee4qameabeaaaSqabaGccqGH9aqpdaaeWaqaaiGbc2gaTjabcggaHjabcIha4jabcIcaOiabdkgaInaaBaaaleaacqWGObaAdaqhaaadbaGaemyAaKMaeyOeI0Iaem4AaSgabaGaem4AaSgaaaWcbeaakiabgEna0kabl+UimjabgEna0kabdkgaInaaBaaaleaacqWGObaAdaqhaaadbaGaemyAaKgabaGaem4AaSgaaaWcbeaakiabgkHiTiabigdaXiabcYcaSiabigdaXiabcMcaPaWcbaGaemyAaKMaeyypa0JaeGymaedabaGaeiiFaWNaemiAaG2aaWbaaWqabeaacqWGRbWAaaWccqGG8baFa0GaeyyeIuoaaaaa@8ED0@

where hi−kk=h|hk|−i+kk
 MathType@MTEF@5@5@+=feaafiart1ev1aaatCvAUfKttLearuWrP9MDH5MBPbIqV92AaeXatLxBI9gBaebbnrfifHhDYfgasaacH8akY=wiFfYdH8Gipec8Eeeu0xXdbba9frFj0=OqFfea0dXdd9vqai=hGuQ8kuc9pgc9s8qqaq=dirpe0xb9q8qiLsFr0=vr0=vr0dc8meaabaqaciaacaGaaeqabaqabeGadaaakeaacqWGObaAdaqhaaWcbaGaemyAaKMaeyOeI0Iaem4AaSgabaGaem4AaSgaaOGaeyypa0JaemiAaG2aa0baaSqaaiabcYha8jabdIgaOnaaCaaameqabaGaem4AaSgaaSGaeiiFaWNaeyOeI0IaemyAaKMaey4kaSIaem4AaSgabaGaem4AaSgaaaaa@41A7@ if *i *- *k *≤ 0; EDFkL
 MathType@MTEF@5@5@+=feaafiart1ev1aaatCvAUfKttLearuWrP9MDH5MBPbIqV92AaeXatLxBI9gBaebbnrfifHhDYfgasaacH8akY=wiFfYdH8Gipec8Eeeu0xXdbba9frFj0=OqFfea0dXdd9vqai=hGuQ8kuc9pgc9s8qqaq=dirpe0xb9q8qiLsFr0=vr0=vr0dc8meaabaqaciaacaGaaeqabaqabeGadaaakeaacqqGfbqrcqqGebarcqqGgbGrdaWgaaWcbaGaee4AaS2aaSbaaWqaaiabbYeambqabaaaleqaaaaa@32BF@ and EDFkC
 MathType@MTEF@5@5@+=feaafiart1ev1aaatCvAUfKttLearuWrP9MDH5MBPbIqV92AaeXatLxBI9gBaebbnrfifHhDYfgasaacH8akY=wiFfYdH8Gipec8Eeeu0xXdbba9frFj0=OqFfea0dXdd9vqai=hGuQ8kuc9pgc9s8qqaq=dirpe0xb9q8qiLsFr0=vr0=vr0dc8meaabaqaciaacaGaaeqabaqabeGadaaakeaacqqGfbqrcqqGebarcqqGgbGrdaWgaaWcbaGaee4AaS2aaSbaaWqaaiabboeadbqabaaaleqaaaaa@32AD@ are for linear and circular chains, respectively. The total EDF for a mixture Markov model is just the sum of EDFs of all individual chains. For example, the total EDF for the special 0-k mixture model equals to the EDF sum of the 0^*th *^and the *k*^*th *^order chains.

### Performance assessment

We test the effectiveness of our method on TFBS data and the donor splice sites, where training data for OMiMa are a set of sequences of a motif. For prediction results, we use the following abbreviations for empirical quantities: *TP *(# true positives), *TN *(# true negatives), *FP *(# false positives), *FN *(# false negatives), *Ac *(Accuracy), *Sn *(sensitivity), *Sp *(specificity), and *Mc *(Matthews correlation coefficient). *Sn, Sp, Ac*, and *Mc *are defined as:

Sn=TPTP+FNSp=TNTN+FPAc=TP+TNTP+FP+TN+FNMc=TP×TN−FP×FN(TP+FN)(TP+FP)(TN+FP)(TN+FN)
 MathType@MTEF@5@5@+=feaafiart1ev1aaatCvAUfKttLearuWrP9MDH5MBPbIqV92AaeXatLxBI9gBaebbnrfifHhDYfgasaacH8akY=wiFfYdH8Gipec8Eeeu0xXdbba9frFj0=OqFfea0dXdd9vqai=hGuQ8kuc9pgc9s8qqaq=dirpe0xb9q8qiLsFr0=vr0=vr0dc8meaabaqaciaacaGaaeqabaqabeGadaaakeaafaqaaeabbaaaaeaacqWGtbWucqWGUbGBcqGH9aqpdaWcaaqaaiabdsfaujabdcfaqbqaaiabdsfaujabdcfaqjabgUcaRiabdAeagjabd6eaobaaaeaacqWGtbWucqWGWbaCcqGH9aqpdaWcaaqaaiabdsfaujabd6eaobqaaiabdsfaujabd6eaojabgUcaRiabdAeagjabdcfaqbaaaeaacqWGbbqqcqWGJbWycqGH9aqpdaWcaaqaaiabdsfaujabdcfaqjabgUcaRiabdsfaujabd6eaobqaaiabdsfaujabdcfaqjabgUcaRiabdAeagjabdcfaqjabgUcaRiabdsfaujabd6eaojabgUcaRiabdAeagjabd6eaobaaaeaacqWGnbqtcqWGJbWycqGH9aqpdaWcaaqaaiabdsfaujabdcfaqjabgEna0kabdsfaujabd6eaojabgkHiTiabdAeagjabdcfaqjabgEna0kabdAeagjabd6eaobqaamaakaaabaGaeiikaGIaemivaqLaemiuaaLaey4kaSIaemOrayKaemOta4KaeiykaKIaeiikaGIaemivaqLaemiuaaLaey4kaSIaemOrayKaemiuaaLaeiykaKIaeiikaGIaemivaqLaemOta4Kaey4kaSIaemOrayKaemiuaaLaeiykaKIaeiikaGIaemivaqLaemOta4Kaey4kaSIaemOrayKaemOta4KaeiykaKcaleqaaaaaaaaaaa@8706@

Matthews correlation coefficient [30], also called Phi (correlation) coefficient, has a value between -1 and 1, with 1, 0, and < 0 indicating a perfect prediction, a random prediction, and a worse than random prediction, respectively.

OMiMa can use two ways to score a motif site **x**: log-likelihood and log-likelihood ratio, which are defined by

log⁡−likelihood=log⁡Pr⁡(x|Ms)log⁡−likelihood ratio=log⁡Pr⁡(x|Ms)Pr⁡(x|Mb)
 MathType@MTEF@5@5@+=feaafiart1ev1aaatCvAUfKttLearuWrP9MDH5MBPbIqV92AaeXatLxBI9gBaebbnrfifHhDYfgasaacH8akY=wiFfYdH8Gipec8Eeeu0xXdbba9frFj0=OqFfea0dXdd9vqai=hGuQ8kuc9pgc9s8qqaq=dirpe0xb9q8qiLsFr0=vr0=vr0dc8meaabaqaciaacaGaaeqabaqabeGadaaakeaafaqadeGabaaabaGagiiBaWMaei4Ba8Maei4zaCMaeyOeI0IaeeiBaWMaeeyAaKMaee4AaSMaeeyzauMaeeiBaWMaeeyAaKMaeeiAaGMaee4Ba8Maee4Ba8MaeeizaqMaeyypa0JagiiBaWMaei4Ba8Maei4zaCMagiiuaaLaeiOCaiNaeiikaGccbeGae8hEaGNaeiiFaWNaemyta00aaSbaaSqaaiabdohaZbqabaGccqGGPaqkaeaacyGGSbaBcqGGVbWBcqGGNbWzcqGHsislcqqGSbaBcqqGPbqAcqqGRbWAcqqGLbqzcqqGSbaBcqqGPbqAcqqGObaAcqqGVbWBcqqGVbWBcqqGKbazcqqGGaaicqqGYbGCcqqGHbqycqqG0baDcqqGPbqAcqqGVbWBcqGH9aqpcyGGSbaBcqGGVbWBcqGGNbWzdaWcaaqaaiGbccfaqjabckhaYjabcIcaOiab=Hha4jabcYha8jabd2eannaaBaaaleaacqWGZbWCaeqaaOGaeiykaKcabaGagiiuaaLaeiOCaiNaeiikaGIae8hEaGNaeiiFaWNaemyta00aaSbaaSqaaiabdkgaIbqabaGccqGGPaqkaaaaaaaa@81E4@

where *M*_*s *_is the signal model trained by true motif sites, and *M*_*b *_is the background model or false signal model trained by background sequences or false motif sites. A sequence **x** is predicted as a positive site if the score of **x** is larger than a certain threshold. We select a cutoff threshold using one of the following three criteria: balanced sensitivity and specificity, the maximum prediction accuracy, and the maximum Matthews correlation coefficient. Each potential threshold yields an estimated true positive rate and a false positive rate. The plot of true positive rates against false positive rates generates a Receiver Operating Characteristic (ROC) curve, which can be used for comparing and selecting the best model.

We used a three-symbol notation 'k-m-s' to distinguish different models, where 'k' stands for a 0-k mixture Markov model, 'm' is either 'L' or 'C' to indicate whether the *k*^*th *^order chain is linear ('L') or circular ('C'), and 's' is either 0 or 1 to indicate whether log likelihood score (0) or log-likelihood ratio score (1) is used. For example, '1-L-l' stands for a 0–1 mixture of linear Markov models that uses log-likelihood ratio to score a motif site.

### Effectiveness of DNJ method for optimization

To assess the ability of our DNJ method for optimizing a Markov chain, we compared the DNJ method with random permutation method. In this evaluation, we used a 0-k mixture model (*k *= 0, 1, 2) (Figure 2) to model transcription factor binding sites from the Transfac database. For each TFBS, we first fitted a 0-k mixture model (denoted as *M*_*DNJ*_) with its *k*^*th *^order Markov chain optimized by the DNJ method. We calculated the log-likelihood of the data given the model *M*_*DNJ *_(log Pr(*data*|*M*_*DNJ*_)). Second, with the same data, we fitted a new 0-k mixture model (denoted as *M*_*R*_), which is the same as *M*_*DNJ *_except that the positions in its *k*^*th *^order chain are ordered by random permutation, and calculated logPr(*data*|*M*_*R*_). This step was repeated 1,000 times, so we have 1,000 log-likelihoods of the randomly permuted models (MR1,…,MR1000)
 MathType@MTEF@5@5@+=feaafiart1ev1aaatCvAUfKttLearuWrP9MDH5MBPbIqV92AaeXatLxBI9gBaebbnrfifHhDYfgasaacH8akY=wiFfYdH8Gipec8Eeeu0xXdbba9frFj0=OqFfea0dXdd9vqai=hGuQ8kuc9pgc9s8qqaq=dirpe0xb9q8qiLsFr0=vr0=vr0dc8meaabaqaciaacaGaaeqabaqabeGadaaakeaacqGGOaakcqWGnbqtdaWgaaWcbaGaemOuai1aaSbaaWqaaiabigdaXaqabaaaleqaaOGaeiilaWIaeSOjGSKaeiilaWIaemyta00aaSbaaSqaaiabdkfasnaaBaaameaacqaIXaqmcqaIWaamcqaIWaamcqaIWaamaeqaaaWcbeaakiabcMcaPaaa@3B66@. We then calculated the empirical *p_value *of the DNJ-optimized model as follows:

p_value=∑i=11000δ(log⁡Pr⁡(data|MDNJ)<log⁡Pr⁡(data|MRi))1000
 MathType@MTEF@5@5@+=feaafiart1ev1aaatCvAUfKttLearuWrP9MDH5MBPbIqV92AaeXatLxBI9gBaebbnrfifHhDYfgasaacH8akY=wiFfYdH8Gipec8Eeeu0xXdbba9frFj0=OqFfea0dXdd9vqai=hGuQ8kuc9pgc9s8qqaq=dirpe0xb9q8qiLsFr0=vr0=vr0dc8meaabaqaciaacaGaaeqabaqabeGadaaakeaacqWGWbaCcqGHsislcqWG2bGDcqWGHbqycqWGSbaBcqWG1bqDcqWGLbqzcqGH9aqpdaWcaaqaamaaqadabaacciGae8hTdqMaeiikaGIagiiBaWMaei4Ba8Maei4zaCMagiiuaaLaeiOCaiNaeiikaGIaemizaqMaemyyaeMaemiDaqNaemyyaeMaeiiFaWNaemyta00aaSbaaSqaaiabdseaejabd6eaojabdQeakbqabaGccqGGPaqkcqGH8aapcyGGSbaBcqGGVbWBcqGGNbWzcyGGqbaucqGGYbGCcqGGOaakcqWGKbazcqWGHbqycqWG0baDcqWGHbqycqGG8baFcqWGnbqtdaWgaaWcbaGaemOuai1aaSbaaWqaaiabdMgaPbqabaaaleqaaOGaeiykaKIaeiykaKcaleaacqWGPbqAcqGH9aqpcqaIXaqmaeaacqaIXaqmcqaIWaamcqaIWaamcqaIWaama0GaeyyeIuoaaOqaaiabigdaXiabicdaWiabicdaWiabicdaWaaaaaa@6F73@

where *δ *is an indicator function with value 1 if condition is true, and 0 otherwise. The smaller the *p_value*, the better the DNJ optimization is; and *p_value *= 0 means the DNJ-optimized model performs better than any one of the 1,000 randomly permuted models. The *p_value *approximates the probability of observing log Pr(*date*|MRi
 MathType@MTEF@5@5@+=feaafiart1ev1aaatCvAUfKttLearuWrP9MDH5MBPbIqV92AaeXatLxBI9gBaebbnrfifHhDYfgasaacH8akY=wiFfYdH8Gipec8Eeeu0xXdbba9frFj0=OqFfea0dXdd9vqai=hGuQ8kuc9pgc9s8qqaq=dirpe0xb9q8qiLsFr0=vr0=vr0dc8meaabaqaciaacaGaaeqabaqabeGadaaakeaacqWGnbqtdaWgaaWcbaGaemOuai1aaSbaaWqaaiabdMgaPbqabaaaleqaaaaa@30BB@) larger than (log Pr(*data*|*M*_*DNJ*_).

Fifty-three human transcription factors, whose binding sites contain at least four dependent positions by the *χ*^2 ^test given by equation (3), are selected for this evaluation (Table 1). The assessment was performed on four 0-k mixture models: 1^*st *^order linear chain, 1^*st *^order circular chain, 2^*nd *^order linear chain, and 2^*nd *^order circular chain.

**Figure 4:**
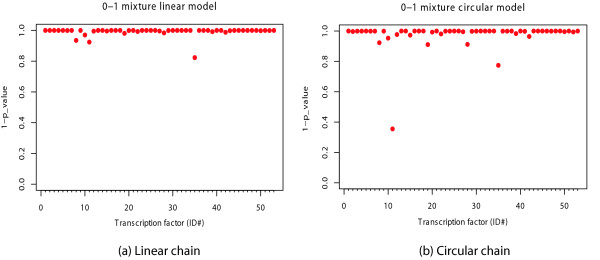
**The performance of the DNJ optimized 0–1 mixture models**. The performance of the DNJ optimized 0–1 mixture models of TFBS. The y-axis is 1-*p_value *measuring performance of the DNJ optimized models relative to the randomly permuted models. The values on x-axis are the ID numbers of 53 TFBS in the first column of Table 1. (a) 0–1 mixture linear models, (b) 0–1 mixture circular models.

Results suggest that DNJ method performed remarkably well in optimizing the 1^*st *^order linear Markov chains, that in 49 out 53 cases, the DNJ optimized models were the best or close to the best (Figure 4a). The optimization for the 2^*nd *^order linear chains was slightly worse than that for the 1^*st *^order linear chains, partially because the DNJ method relies only on the pairwise dependencies between two single positions. Nevertheless, most of the DNJ optimized models were still close to the best [see Additional file 1 Figure 1a]. Although our DNJ method was designed for optimizing linear Markov chains, it still worked well in optimizing the 1^*st *^order circular Markov chains (Figure 4b). However, the DNJ method did not perform well in optimizing the 2^*nd *^order circular Markov chains [see Additional file 1 Figure 1b].

**Figure 5:**
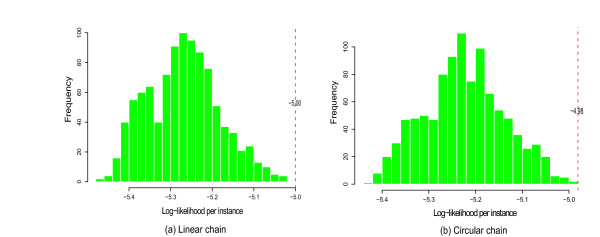
**Modeling TFBS V$AP1_Q4_01**. The performance of the optimized model of TFBS V$AP1_Q4_01. The histogram is the log-likelihood score distribution of 1,000 randomly permuted mixture models. The red reference line indicates the relative performance of the DNJ optimized model (a) 0–1 mixture linear model (b) 0–1 mixture circular model.

We used AP1 (activating protein 1) transcription factor binding sites (Transfac ID V$AP1_Q4_01) as an example of how DNJ optimization can improve performance of a 0–1 or 0–2 mixture model. We plotted the histogram of the log-likelihood per instance given a model MRi
 MathType@MTEF@5@5@+=feaafiart1ev1aaatCvAUfKttLearuWrP9MDH5MBPbIqV92AaeXatLxBI9gBaebbnrfifHhDYfgasaacH8akY=wiFfYdH8Gipec8Eeeu0xXdbba9frFj0=OqFfea0dXdd9vqai=hGuQ8kuc9pgc9s8qqaq=dirpe0xb9q8qiLsFr0=vr0=vr0dc8meaabaqaciaacaGaaeqabaqabeGadaaakeaacqWGnbqtdaWgaaWcbaGaemOuai1aaSbaaWqaaiabdMgaPbqabaaaleqaaaaa@30BB@, log Pr(*data*|MRi
 MathType@MTEF@5@5@+=feaafiart1ev1aaatCvAUfKttLearuWrP9MDH5MBPbIqV92AaeXatLxBI9gBaebbnrfifHhDYfgasaacH8akY=wiFfYdH8Gipec8Eeeu0xXdbba9frFj0=OqFfea0dXdd9vqai=hGuQ8kuc9pgc9s8qqaq=dirpe0xb9q8qiLsFr0=vr0=vr0dc8meaabaqaciaacaGaaeqabaqabeGadaaakeaacqWGnbqtdaWgaaWcbaGaemOuai1aaSbaaWqaaiabdMgaPbqabaaaleqaaaaa@30BB@)/*N*, where *N *is the number of sequences in the data set, and *i *= 1,..., 1000 for 1,000 mixture models of randomly permuted Markov chains. The histogram represents a simulated null-distribution of log-likelihood per instance given a mixture model. Then we mapped the location of the likelihood per instance given DNJ optimized model, (log Pr(*data*|*M*_*DNJ*_)/*N*, in the histogram. For the transcription factor V$AP1_Q4_01, we found that for the 0–1 mixture model of either linear or circular structure, DNJ optimized models are better than any models from 1,000 random permutations (Figure 5).

Theoretically, the optimal model can be found by exhaustively searching through all possible models. An exhaustive search is not always possible in practice, however, as the search space can be very large. The number of possible Markov chains is the factorial of the length of the Markov chain and dramatically increases as the length of chain increases. For example, the computational time for a motif of 15 bases (15! = 1.307674e + 12) can be practically unacceptable. Our DNJ method can deal with such long motifs because of its computational efficiency.

### TFBS identification

One interesting application of our mixture model is TFBS identification. In this assessment, we used a couple of examples to show how OMiMa can improve prediction accuracy when there are position dependencies within a TFBS. We first tested our method on simulated data where the exact dependency structure of a TFBS is known. We tested whether OMiMa can capture such dependency and optimize the Markov model accordingly. Next, we tested our method on real motif data for AP1. In both examples, we compared OMiMa performance to PWM, PVLMM, and the 1^*st *^order Markov model (1stMM) with its motif positions in the natural order. PVLMM, run on Microsoft Windows, is based on the variable length Markov model (VLMM) [31,32]. Except for its order and depth parameters, PVLMM was run under its default settings in all comparisons.

#### Simulated TFBS prediction

Many TFBS are palindromic sites bound by heterodimers/homodimers (*e.g*.,: Jun-Fos, Myc-Max, Max-Max and p50-p50). The sequences in two half sites of a palindromic TFBS are usually not perfectly complementary, and the strong binding to one half site may compensate for weak binding to the other. We simulated two imperfect palindromic TFBS (named A and B) of length 12 bases. For each TFBS, the bases in each position were generated from the uniform distribution (the frequency of each base is 0.25). The base in one half site and its reverse complementary base in the other half were generated using the probabilities listed in Table 2. Therefore, there are only pairwise position dependencies in the simulated TFBS. The position pair 0–11 has the strongest dependency, whereas the pair 5–6 has the weakest dependency (they are independent). Overall, motif A has stronger position dependencies than motif B. The false sites of TFBS were simulated from the uniform distribution of four nucleotides without any constraints of base pairing between the two half sites. The simulated data of each TFBS consist of a training set with 150 true sites, and a testing set with 150 true sites and 150 false sites. Using these simulated training sets, OMiMa found all true dependencies significant at level *α *= 0.05 (see Table 2). For both TFBS, OMiMa was also able to arrange the positions of each dependent pair to be the nearest neighbors in their 0–1 mixture models: (See table 7)

**Figure 6:**
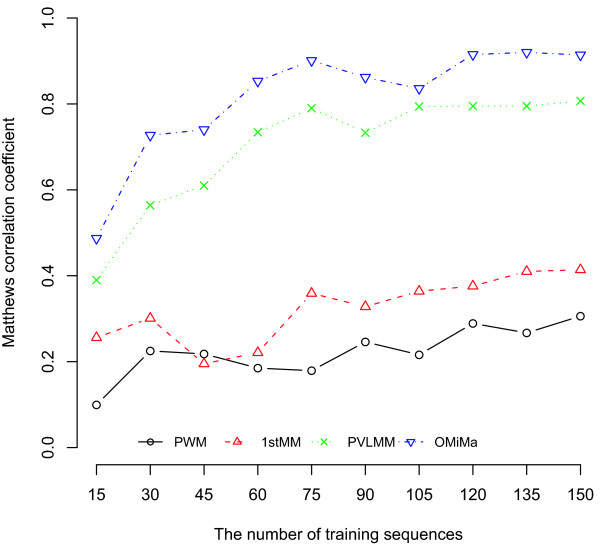
**Performance comparison on the simulated palindromic TFBS**. The performance comparison of different methods for predicting the simulated palindromic TFBS A. The x-axis shows the number of motif sequences used for training. The y-axis is the Matthews correlation coefficient of each method in predicting the same testing dataset (150 false and 150 true sites, respectively). The figure shows that OMiMa performed significantly better than the other methods, regardless the number of training samples.

In our simulation, the positions 5 and 6 were generated independently from all other positions, so they should be in the 0^*th *^order chains. However, based on OMiMa's pairwise *χ*^2 ^tests for the training data, the position pair 5–8 (with p-value = 0.03) in TFBS A, and the position pair 6–10 (with p-value = 0.04) in TFBS B were declared dependent. That is why the positions 5 and 8 were arranged together in the model for TFBS A, and positions 6 and 10 were together for TFBS B. We compared the prediction results of OMiMa's 0–1 mixture model with those of PWM, 1stMM and the 1^*st *^order PVLMM (with depth 1). Results (Table 3) showed that OMiMa outperformed all other models, and PVLMM performed better than IstMM and PWM. Additionally, we used smaller training sets to access the performance of these methods on the same testing set. Smaller training sets, in sizes ranging from 15 to 150, were independently sampled (without replacement) from the original 150 sites for training. Results suggested that OMiMa performed consistently better than the other methods, regardless the size of a training set (Figure 6) [see Additional file 1 Figure 2].

#### AP1 TFBS prediction

**Figure 7:**
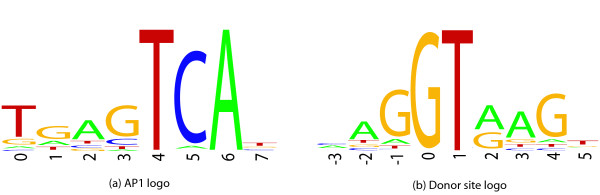
**The sequence logos of AP1 TFBS and the donor site**. Sequence logos of the AP1 TFBS and the donor splice site. The height of bases represents the information content at each position of a sequence motif. (a) the logo of AP1 TFBS. Note that the positions 4 and 6 of AP1 TFBS are not perfectly conserved. (b) the logo of donor splice site. The positions 0 and 1 are perfectly conserved. The logo plot was created by WebLogo [45].

We chose human AP1 TFBS (see Figure 7a) for this evaluation because of its relatively large number of known sites. In total, we had 119 true sites and 5950 false sites. The true sites were extracted from Transfac database (Transfac ID V$AP1_Q4_01), and false sites were randomly sampled from the non-coding regions of the human genome. Our *χ*^2 ^tests on the 119 true sites suggested that all positions showed some level of dependency with the neighboring pairs 0–2, 4–5, 5–6, and 4–6 showing strong dependencies (p-value < 1.0e-6). Noticeably, the positions 4, 5 and 6 are also the most conserved positions, so we expect that PWM would be reasonable good model for the TFBS. We randomly split both the true sites and false sites into 10 roughly equal-sized parts, and used a 10-fold cross validation to compare the performance of OMiMa's 0–1 mixture model with the others. OMiMa had advantage over the other three models in predicting TFBS that do not have strong long-range dependencies (Table 4). Results also showed the first order PVLMM did not perform better than 1stMM and PWM. We found that the first order PVLMM arranged the position pair 5–6, which showed the strong dependency, differently from OMiMa and 1stMM. In only 3 out 10 times, PVLMM arranged positions 5 and 6 as direct neighbors, while OMiMa did in 9 out 10 times, and 1stMM did naturally all times. This is one possible reason why PVLMM performed slightly worse.

### Donor splice site recognition

The transcription of most higher eukaryotic genes involves RNA splicing, in which primary transcripts become mature mRNA by removing introns. The donor or 5' splice sites and the acceptor or 3' splice sites on the boundaries of exons and introns provides critical signals for precise splicing. Therefore, splice site recognition has been widely used by gene finding tools such as GENESCAN [2] and GENIE [33] for gene prediction. The splicing process starts with U1 snRNP binding to the donor site via base-pairing of U1 snRNA. The base pairing between U1 snRNA and the donor site, however, need not be perfectly complementary in all positions [34,35]. Both experimental and computational evidence suggest that there are mutually dependent positions within the donor site: a mis-matched pair of U1 snRNA and the donor sites at one position can be compensated for by a matching base pair at another position, and vice versa [2,24,36,37]. Modeling such dependency structure within the donor site has been used to improve donor site prediction [2,23,24,33]. We used two independent datasets of human donor sites to assess the performance of OMiMa in comparison with leading competitors.

#### Comparison with NNSplice and PVLMM

The test dataset of human donor splice sites (Reese data) was from [38]. This dataset has 6246 donor sites (1324 real and 4922 false) of length 15 bases from -7 to +8 around the conserved 'GT' dinucleotide. The dataset consists of a training set (containing 56
 MathType@MTEF@5@5@+=feaafiart1ev1aaatCvAUfKttLearuWrP9MDH5MBPbIqV92AaeXatLxBI9gBaebbnrfifHhDYfgasaacH8akY=wiFfYdH8Gipec8Eeeu0xXdbba9frFj0=OqFfea0dXdd9vqai=hGuQ8kuc9pgc9s8qqaq=dirpe0xb9q8qiLsFr0=vr0=vr0dc8meaabaqaciaacaGaaeqabaqabeGadaaakeaadaWcaaqaaiabiwda1aqaaiabiAda2aaaaaa@2EAE@ of data) and a testing set (the remainder), which were previously used to assess the performance of NNSplice [33]. We used the same partitions for training and testing in the following comparisons.

**Figure 8:**
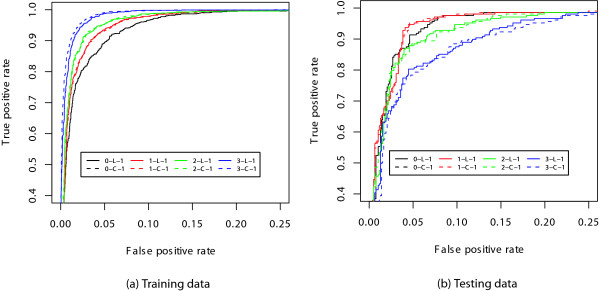
**Comparison of different 0-k mixture models for donor splice site prediction**. Comparison of different 0-k mixture models for donor splice site prediction by ROC curves. Based on the Area Under Curve (AUC) criterion, the figure indicates that: (a) For training data, the best models were 3-L-1 and 3-C-1 while the worst model is 0-L-1 (same as 0-C-1). (b) For testing data, the best models were 1-L-1 and 1-C-1 while the worst models are 3-L-1 and 3-C-1.

First, we tested whether OMiMa which uses either AIC or BIC, can correctly pick the best model based on ROC analysis. We fitted a set of 0-k mixture models, in which the *k*^*th *^order chains are either linear or circular and *k *ranges from 0 to 3, with the training data. We subsequently applied the fitted models to predict splice sites in testing data. The performances of different models were compared and evaluated by ROC analysis (Figure 8). In addition, we compared the maximum accuracy (*Ac*) and the maximum Matthews correlation efficient (*Mc*) achieved by each model (data not shown). The best models were 0–1 mixture models (Figure 8). Both the linear and circular models performed about the same. The best models picked by ROC analysis are consistent with those selected by OMiMa (AIC criterion). The selected models were further confirmed by a six-fold cross validation.

Using the best model selected above, we then compared OMiMa with NNSplice and PVLMM. NNSplice is based on a complex neural network model and is trained by both true sites and false sites. Since both OMiMa and NNSplice used the same training and testing data, their prediction results can be directly compared. We compared OMiMa's 1-L-1 and 1-C-1 models with the first order PVLMM (with depth 1) as all have similar model complexity. The results of NNSplice were reported at the NNSplice Web site [39]. We found that OMiMa had comparable prediction accuracy to NNSplice and PVLMM (Table 5). In addition, OMiMa is much more computationally efficient than NNSplice and PVLMM [see Additional file 1].

#### Comparison with MEM and PVLMM

Given enough training data, we can use more complicated models than the 0–1 mixture model to improve prediction accuracy. In this evaluation, we test whether 0-k mixture models can compete with the MEM on a much larger dataset. This large donor site dataset (Yeo data), used to assess performance of the MEM, was from [40]. The dataset, extracted from 1821 non-redundant human transcripts, has 8,415 real and 179,438 decoy sites in the training set, and 4,208 real and 89,717 in decoy sites in the testing set. Each real site has length 9 bases from -3 to +6 around the conserved 'GT' of donor splice sites recognized by U-2 type spliceosome. The decoy sites are any other sequence segments in the exons and introns matching the pattern *N*_3_*GTN*_4_. So a decoy site can have the exactly same sequence as a real site. We applied this original training and testing sets to assess performance of OMiMa, where we used only log-likelihood ratio scoring. In addition, we ran a 3-fold cross-validation, in which the number of sites in new training and testing sets are roughly the same as those in the original ones [see Additional file 1 Table 2]. The top 4 models selected by AIC are 3-C-1, 3-L-1, 2-C-1 and 2-L-1, respectively, consistent with the ROC analysis. To find the top 4 sub-models of PVLMM by ROC analysis, we used a series of Markov orders and/or depths (1 ≤ *order *≤ 4 and *order *≥ *depth*) to predict the same data sets. For convenience, we use notation "P:*k*-*d*" to denote a PVLMM of order *k *and depth *d*. We adopt notation in [23] for sub-models of MEM.

Briefly, the notation has the form "me*K*s*D*" or "me*K*x*D*", where "me" stands for maximum entropy; "*K*" is a number for the marginal order or the maximum length of an oligomer in consideration; "*D*" is the skip number or maximum skip number determining which positions the bases of an oligomer are from; "s" stands for skip number and "x" for the maximum skip. For example, model "me5s0" considers all marginal distributions of *p*(*x*_*i*_), *p*(*x*_*i*_, *x*_*i*+1_), *p*(*x*_*i*_, *x*_*i*+1_, *x*_*i*+2_), *p*(*x*_*i*_, *x*_*i*+1_, *x*_*i*+2_, *x*_*i*+3_), *p*(*x*_*i*_, *x*_*i*+1_, *x*_*i*+2_, *x*_*i*+3_, *x*_*i*+4_).

Comparison of the top 4 performers from each model class suggested that OMiMa performed comparably with MEM and better than PVLMM (Table 6) (all models' performances suffered on this data set because about 98% real sites appeared at least once in the decoy set). One advantage of OMiMa over MEM is that, for the models with similar performance, OMiMa's models generally have fewer parameters and thus require fewer training samples. Our test showed that OMiMa was able to retain similar performance of MEM even when trained by only 60% data of MEM's original training sets [see Additional file 1 Table 3].

#### Biological explanation

To compare OMiMa's fitted donor site models to biological knowledge about dependencies among positions, we examined the best donor models for the first donor dataset (Reese data) and for the second donor dataset (Yeo data). For convenience, let us mark the invariant 'GT' nucleotides in the boundary of exon/intron as the positions 0 and 1 of the donor site, respectively (see Figure 7b). First, based on 1,116 real donor sites in the Reese original training data, the 0–1 mixture model was selected as the best model with the following 1^*st *^order chain.

-2 5 -1 3 4 -3 -7 -6 -5 -4 7 6 2

We found that this position arrangement is supported by the following biological evidence of base-pairing between U1 snRNA and the donor site: (a) 5'/3' compensation effect: a base pair at position -1 can prevent an aberrant splicing caused by a mis-matched pair at position 5 [37]; (b) Adjacent base-pair effect: a matching base pair at position 3 is rare in the absence of a matching base pair at position 4 [2,41]; (c) A matching base-pair at the non-conserved positions 6 and 7 can compensate for a mis-matched pair at position 2 [42]. Interestingly, the model also arranged non-conserved positions (-4, -5, -6, -7, 6, 7) together as it did for the other more conserved positions. Second, based on 8,415 real donor sites of the Yeo original training sites, the 0–3 mixture model was the best model. The optimized position order of its 3^*rd *^order chain was:

2 5 -1 4 -2 3 -3

We can see that this model is consistent with the above evidence (a) and (b). In addition, it is well supported by experimentally verified position dependencies of position 4 on the positions -1, -2, 3 and 5 [37], and the computationally confirmed dependency of position -3 on position -2 due to the adjacent base-pair effect [2].

## Discussion

The prediction accuracy of a probabilistic model is largely determined by the effectiveness of the model in characterizing a biological motif. Since there is large variation of the signals embedded in biological motifs, an effective model can be as simple as a consensus sequence or as complex as a fully connected network model. In this paper, we described a mixture of Markov models to allow adjustment of model complexity for different motifs. Also, we extended the traditional linear chain Markov model to the circular chain Markov model, which can better represent position dependencies within a motif in some cases. We presented a novel method, DNJ, for efficiently optimizing position arrangement of a non 0^*th *^order Markov chain to incorporate most dependencies. We described methods for calculating the EDF and for selecting the best mixture Markov model. We implemented these methods in our motif finding OMiMa system, which is freely available. Finally, we demonstrated from different aspects in several examples that OMiMa can improve motif prediction accuracy in biological sequences.

The interaction of biological macromolecules, such as transcription factors bound to DNA sites, usually involves several highly dependent positions functioning as a unit. Many methods including Markov chains, Bayesian trees, and neutral networks have been used to model dependency structures within a motif. The Markov model is the simplest yet can be very powerful when it is optimized. Our results showed that the optimized Markov models performed better than the neural network model and PVLMM, and comparably with MEM for splice site prediction. The optimized Markov model can incorporate both local and non-local dependencies into the model, which enables it to compete with tree or network models in predicting short biological motifs. We also showed that the optimized Markov model can be an excellent motif predictor. Moreover, it is also computationally efficient due to its simplicity.

Model complexity, measured by parameter number, is an important issue in motif modeling. The more complex a model, the more data are needed for adequate training. For many biological motifs, however, the number of known (experimentally determined) sites is small. This limits the usage of complex models, such as higher order Markov models, Bayesian trees, network models or MEM, even though these models in some cases can perform better than the simpler models given enough training data. For a standard Markov model, the number of its parameters increases exponentially as its Markov order increases. Without sufficient training data, it is difficult to accurately estimate all model parameters, even using more robust methods (*e.g*. interpolated Markov chains [43,44]). As a result, lack of sufficient training data often makes it impractical to train a higher order Markov model. On the other hand, a low order Markov model may perform poorly by failing to incorporate more distant dependencies. Several motif models and methods have been developed to address this issue. One of these models is the variable length Markov model (VLMM), whose Markov orders (also called context lengths) can vary among different positions. VLMM can effectively reduce Markov model complexity when the variation of actual context lengths is large. VLMM, however, is not the best choice to incorporate long-range dependencies. The position optimized Markov model (POMM) [21] is able to incorporate important distant dependencies without increasing Markov chain order. However, the effectiveness of this model largely depends on the optimization routine.

More recently, Zhao *et al. *[24] described the PVLMM in an attempt to combine advantages of both VLMM and POMM. The disadvantage of PVLMM is that the number of possible permutations is the factorial of motif length, which makes it more computationally expensive. In addition, the random permutation method used by PVLMM for optimization is more likely to overfit the model, *e.g*., incorporating non-significant dependencies into the PVLMM model that can reduce its prediction power. The optimized mixture of Markov models we presented here tries to inherit advantages of these existing models while avoiding their disadvantages. In OMiMa, we replace VLMM with a mixture of several lower order Markov models, which are subsequently optimized to account for long-range dependencies.

In comparison with other leading methods, OMiMa can incorporate more than the NNSplice's pairwise dependencies; OMiMa avoids model over-fitting better than the PVLMM; and OMiMa requires smaller training samples than the MEM. These are primarily reasons that OMiMa showed superior performance, in terms of prediction accuracy, required size of training data or computational time, over other leading-methods in our results.

With any model selection procedure, the possibility of choosing a model that drastically over- or underfits is a concern. OMiMa employs AIC and BIC, two standard criteria, that are widely used because they tend to avoid extreme over- or underfitting. Both have theoretical support [27,28]. In our application, neither criterion worked well when using the DF (results not shown); but both, particularly AIC, performed well when using EDF. We found that models selected by AIC using EDF were consistent with models selected by cross-validation and by ROC analysis.

Our OMiMa approach has two features that can be limitations when the size of the training data is small. First, the chi-square test that partitions motif positions into those with dependencies and those without dependencies will, like any statistical test, make mistakes, and its statistical power to detect dependencies will suffer with small training samples. Although the test will not always provide a correct partition, our approach should adapt to strong or weak dependencies overall and improve prediction when dependencies are strong. In addition, weakly dependent positions mistakenly placed in the set with no dependencies are often adequately modeled by a 0^*th *^order chain, whereas independent positions mistakenly assigned to the set with dependencies will be placed by the DNJ algorithm in locations with the least impact on the *k*^*th *^order chain. Second, the EDF that we used in model selection is an estimate based on the training data. For degenerate sites, the estimate should be accurate with even small training samples; whereas for conserved sites a larger training sample might reveal additional bases and change the EDF. Still, such additions should be minimal and would generally induce small changes in the EDF, so we expect little impact on model selection. Any methods that employ chi-square techniques to test for dependent sites face similar limitations. Nevertheless, OMiMa with its relatively small parameter space should adapt to small training datasets better than many competitors. Of course, any motif finding algorithm would do better with larger training samples.

OMiMa places no limit on the length of sequences that it can scan, and it could be used to find TFBS in any sequenced organism as long as a training motif set is available. The larger the genome evaluated, the more false positives are likely to be declared. Although OMiMa's prediction accuracy will help, other approaches to reducing false positives will be needed. Cross-species comparisons and relative location compared to transcription start sites have been used to reduce false positives and could be used with OMiMa too. Furthermore, OMiMa's ability to accurately and quickly identify splice sites should be easy to incorporate into probabilistic gene-prediction programs where correct prediction of splice sites is critical.

## Conclusion

Our optimized mixture of Markov models represents an alternative to the existing methods for modeling dependent structures within a biological motif. Unlike existing methods, our model is conceptually simple and effective, which has advantages in a large scale motif prediction. In particular, with its ability to minimize model complexity, our method can work effectively even with limited training data. The optimized mixture of Markov models is implemented in our computational tool OMiMa, which can use a variety of mixture models for motif prediction. OMiMa, in which most parameters are configurable, is freely available to all users.

## Authors' contributions

W. Huang provided the principal contributions to the conception and design of this study as well as to its analysis. D. M. Umbach and L. Li contributed to the design of the study and the interpretation of results. All authors contributed to writing and critically revising the manuscript.

## Supplementary Material

Additional file 1 The supplement includes the mathematical formulas for computing the probability of a motif site given a Markov model, the algorithmic pseudo-code for the DNJ method, and the description of the parameter estimation for our model. It also contains supplemental materials for the main results as well as other additional results, such as the application for protein domain identification, the comparison of computational time, and so on. Click here for file

## Figures and Tables

**Table 1:** The optimized 1^*st *^order Markov chains for TFBS. The optimized arrangement of dependent positions within TFBS for the 1^*st *^order Markov model. N and *N*_*D *_are total number of motif positions and the number of positions significantly dependent, respectively.

ID#	Name	N	*N*_*D*_	Position order
1	V$AP1_Q4_01	8	8	7-3-1-2-0-6-5-4
2	V$AP1_Q6_01	9	8	2-3-1-4-5-7-8-6
3	V$AP1_Q2_01	12	9	4-3-5-6-7-10-11-9-1
4	V$CDPCR1_01	10	9	3-4-2-9-6-5-7-8-1
5	V$ATF_01	14	8	1-0-10-9-11-2-13-12
6	V$CHOP_01	13	10	5-4-6-7-9-10-0-8-11-12
7	V$CDPCR3_01	15	10	3-0-1-8-9-13-4-6-2-5
8	V$CDPCR3HD_01	10	5	1-8-9-2-7
9	V$CREB_Q2_01	14	8	1-11-12-0-2-3-9-8
10	V$CREB_Q4_01	11	6	7-6-1-8-9-10
11	V$CREB_Q3	6	4	4-5-1-0
12	V$CEBP_Q3	12	9	8-9-5-6-4-11-3-2-10
13	V$CEBPB_01	14	4	0-13-11-3
14	V$E2F_Q4_01	11	4	1-8-7-0
15	V$E2F_Q6_01	12	8	8-3-7-0-2-11-9-10
16	V$E2F1DP1_01	8	5	3-4-0-6-7
17	V$E2F1DP2_01	8	5	5-6-7-3-4
18	V$E2F4DP1_01	8	4	3-4-0-1
19	V$E2F4DP2_01	8	5	4-3-7-1-0
20	V$ETS_Q4	12	8	11-2-5-10-4-3-0-1
21	V$ELK1_02	14	4	10-11-2-3
22	V$FAC1_01	14	12	12-6-10-11-13-4-9-8-5-1-0-7
23	V$FOXD3_01	12	11	1-3-8-7-9-10-11-2-0-4-6
24	V$FOXO1_02	14	11	8-9-10-12-7-6-2-0-11-1-3
25	V$HNF4_Q6	9	7	4-3-2-6-8-1-7
26	V$HNF1_Q6	18	15	3-11-12-1-4-8-13-5-9-0-6-16-14-2-10
27	V$HNF3_Q6	13	11	1-10-7-5-3-4-12-9-0-2-8
28	V$E2F1DP1RB_01	8	5	1-7-3-0-4
29	V$IRF7_01	18	13	3-2-0-16-15-17-1-7-6-8-14-9-12
30	V$LUN1_01	17	8	8-9-10-7-12-11-14-13
31	V$MZF1_01	8	4	0-1-4-5
32	V$MYC_Q2	7	4	4-5-3-1
33	V$NFAT_Q4_01	10	4	6-8-9-5
34	V$NFKAPPAB_01	10	4	5-7-9-2
35	V$NKX22_01	10	6	9-8-6-1-0-7
36	V$OCT_Q6	11	10	8-2-0-10-5-3-9-6-4-7
37	V$PAX_Q6	11	10	10-6-7-0-9-3-1-5-4-2
38	V$PAX6_01	21	21	15-17-16-18-6-8-19-13-11-3-2-1-0-20-7-10-4-9-5-14-12
39	V$PBX1_02	15	10	6-12-2-0-3-1-11-13-14-4
40	V$RSRFC4_Q2	17	6	6-7-0-13-2-3
41	V$RSRFC4_01	16	8	6-7-9-1-2-13-12-8
42	V$STAT5A_01	15	7	8-12-1-13-0-4-5
43	V$SOX9_B1	14	9	1-13-0-2-11-5-3-10-4
44	V$SRY_01	7	4	4-6-0-1
45	V$SRY_02	12	4	1-3-11-4
46	V$STAT5A_02	24	16	7-12-20-15-16-17-18-19-22-1-21-13-5-6-9-23
47	V$SP1_Q2_01	10	7	7-3-8-0-4-9-5
48	V$SP1_Q4_01	13	13	0-2-11-12-6-1-3-10-9-8-7-4-5
49	V$SP1_Q6_01	10	10	3-5-8-9-0-7-4-2-6-1
50	V$USF_Q6_01	12	8	3-11-4-5-7-2-1-8
51	V$XBP1_01	17	9	13-5-3-4-15-11-10-12-0
52	V$ZID_01	13	8	6-7-4-8-12-10-9-11
53	I$DRI_01	10	7	6-9-8-7-0-1-2

**Table 2:** Simulation of two palindromic TFBS. Simulation of two palindromic TFBS A and B. The first 2 columns are the complementary positions of the palindromic TFBS. The 3^*rd *^and 4^*th *^columns are simulation parameters, which specify the probabilities of forming a complementary base pair. The last 2 columns are the p-values of OMiMa's pairwise *χ*^2 ^tests of position dependency for the simulated data.

Position pair		Complementary Prob.		p-value	
1^*st*^	2^*nd*^	A	B	A	B
0	11	0.99	0.90	4.88e-88	3.84e-63
1	10	0.95	0.85	6.62e-72	2.66e-56
2	9	0.90	0.75	3.84e-69	2.25e-35
3	8	0.65	0.65	1.44e-19	5.89e-24
4	7	0.50	0.50	2.00e-07	3.05e-03
5	6	0.25	0.25	3.35e-01	1.43e-01

**Table 3:** Performance evaluation using simulated palindromic TFBS. Performance comparison of OMiMa (1-L-0) with PWM, 1stMM, and PVLMM (order 1 and depth 1) for predicting two simulated TFBS A and B. The performance was measured as the maximum *Mc *achieved by each model.

Motif	PWM	1stMM	PVLMM	OMiMa
A	0.306	0.414	0.807	0.914
B	0.253	0.428	0.647	0.794

**Table 4:** TFBS V$AP1_Q4_01 prediction. Comparison of OMiMa (1-L-0/1-C-0), PWM, 1stMM, and PVLMM (order 1 and depth 1) for AP1 TFBS prediction. The performance results are the average values of 10-fold cross validation.

Model	*Sn*	*Sp*	*Mc*
PWM	0.857	0.997	0.860
1stMM	0.839	0.998	0.870
PVLMM	0.789	0.999	0.847
1-L-0	0.866	0.998	0.882
1-C-0	0.874	0.998	0.884

**Table 5:** Comparison OMiMa with NNSplice and PVLMM for donor site prediction. Comparing two OMiMa models (1-L-1 and 1-C-1) with NNSplice's neural network model and PVLMM (order 1 and depth 1) for donor splice site prediction.

		Network	PVLMM	1-L-1	1-C-1
*Ac *maximized	*Ac*	0.951	0.927	0.955	0.954
	*Sn*	0.904	0.793	0.928	0.947
	*Sp*	0.963	0.963	0.962	0.955

*Mc *maximized	*Mc*	0.857	0.786	0.869	0.869
	*Sn*	0.942	0.889	0.938	0.952
	*Sp*	0.951	0.934	0.959	0.954

**Table 6:** Comparison of OMiMa PVLMM and MEM for donor site prediction. Comparing OMiMa with PVLMM and MEM for donor splice site prediction. The table shows Matthews correlation efficients (*Mc*) of top 4 models from each model class. The splice site data and results of MEM models were from Yeo and Burge [23]. In the 3-fold cross validation, the sample sizes for both training and testing sets are approximately equal to those of the original partition by Yeo and Burge.

MEM		PVLMM		OMiMa	
sub-model	*Mc*	sub-model	*Mc *(Org./3-CV)	sub-model	*Mc *(Org./3-CV)

me2x5	0.659	P:2-2	0.629/0.631	3-C-1	0.658/0.663
me2x4	0.655	P:3-2	0.626/0.632	3-L-1	0.654/0.657
me2x3	0.653	P:4-2	0.625/0.630	2-C-1	0.647/0.657
me5s0	0.653	P:4-3	0.622/0.628	2-L-1	0.643/0.653

**Table 7:** 

TFBS	0^*th *^chain	1^*st *^chain
A	6	7-4-1-10-3-8-5-0-11-9-2
B	5	2-9-6-10-1-8-3-7-4-11-0

## References

[B1] Weichun Huang's Research Domain. http://BioMedEmpire.org.

[B2] Burge C, Karlin S (1997). Prediction of complete gene structures in human genomic DNA. J Mol Biol.

[B3] Wray GA, Hahn MW, Abouheif E, Balhoff JP, Pizer M, Rockman MV, Romano LA (2003). The Evolution of Transcriptional Regulation in Eukaryotes. Mol Biol Evol.

[B4] Kellis M, Patterson N, Endrizzi M, Birren B, Lander ES (2003). Sequencing and comparison of yeast species to identify genes and regulatory elements. Nature.

[B5] Negre B, Casillas S, Suzanne M, Sanchez-Herrero E, Akam M, Nefedov M, Barbadilla A, de Jong P, Ruiz A (2005). Conservation of regulatory sequences and gene expression patterns in the disintegrating Drosophila Hox gene complex. Genome Res.

[B6] Xie X, Lu J, Kulbokas EJ, Golub TR, Mootha V, Lindblad-Toh K, Lander ES, Kellis M (2005). Systematic discovery of regulatory motifs in human promoters and 3' UTRs by comparison of several mammals. Nature.

[B7] Staden R (1984). Computer methods to locate signals in nucleic acid sequences. Nucl Acids Res.

[B8] Stormo GD, Fields DS (1998). Specificity, free energy and information content in protein-DNA interactions. Trends Biochem Sci.

[B9] Quandt K, Freeh K, Karas H, Wingender E, Werner T (1995). Matlnd and Matlnspector: new fast and versatile tools for detection of consensus matches in nucleotide sequence data. Nucl Acids Res.

[B10] Kel AE, Gössling E, Reuter I, Cheremushkin E, Kel-Margoulis OV, Wingender E (2003). MATCH: A tool for searching transcription factor binding sites in DNA sequences. Nucleic Acids Res.

[B11] Agarwal P, Bafna V (1998). Detecting non-adjoining correlations with signals in DNA. RECOMB '98: Proceedings of the second annual international conference on Computational molecular biology.

[B12] Man TK, Stormo GD (2001). Non-independence of Mnt represser-operator interaction determined by a new quantitative multiple fluorescence relative affinity (QuMFRA) assay. Nucl Acids Res.

[B13] Benos PV, Lapedes AS, Fields DS, Stormo GD (2001). SAMIE: statistical algorithm for modeling interaction energies. Pac Symp Biocomput.

[B14] Bulyk ML, Johnson PLF, Church GM (2002). Nucleotides of transcription factor binding sites exert interdependent effects on the binding affinities of transcription factors. Nucl Acids Res.

[B15] Roulet E, Busso S, Camargo AA, Simpson AJG, Mermod N, Bucher P (2002). High-throughput SELEX SAGE method for quantitative modeling of transcription-factor binding sites. Nat Biotechnol.

[B16] Krivan W, Wasserman WW (2001). A Predictive Model for Regulatory Sequences Directing Liver-Specific Transcription. Genome Res.

[B17] Schneider TD, Stormo GD, Gold L, Ehrenfeucht A (1986). Information content of binding sites on nucleotide sequences. J Mol Biol.

[B18] Zhang MQ, Marr TG (1993). A weight array method for splicing signal analysis. Comput Appl Biosci.

[B19] Ponomarenko MP, Ponomarenko JV, Frolov AS, Podkolodnaya OA, Vorobyev DG, Kolchanov NA, Overton GC (1999). Oligonucleotide frequency matrices addressed to recognizing functional DNA sites. Bioinformatics.

[B20] Cai D, Delcher A, Kao B, Kasif S (2000). Modeling splice sites with Bayes networks. Bioinformatics.

[B21] Ellrott K, Yang C, Sladek FM, Jiang T (2002). Identifying transcription factor binding sites through Markov chain optimization. Bioinformatics.

[B22] Barash Y, Elidan G, Friedman N, Kaplan T (2003). Modeling dependencies in protein-DNA binding sites. RECOMB '03: Proceedings of the seventh annual international conference on Computational molecular biology.

[B23] Yeo G, Burge CB (2004). Maximum entropy modeling of short sequence motifs with applications to RNA splicing signals. J Comput Biol.

[B24] Zhao X, Huang H, Speed TP (2004). Finding short DNA motifs using permuted markov models. RECOMB '04: Proceedings of the eighth annual international conference on Computational molecular biology.

[B25] Zhou Q, Liu JS (2004). Modeling within-motif dependence for transcription factor binding site predictions. Bioinformatics.

[B26] Saitou N, Nei M (1987). The neighbor-joining method: a new method for reconstructing phylogenetic trees. Mol Biol Evol.

[B27] Akaike H (1974). A new look at the statistical model identification. IEEE Trans Automat Control.

[B28] Schwarz G (1978). Estimating the dimension of a model. Ann Stat.

[B29] Wingender E, Chen X, Hehl R, Karas H, Liebich I, Matys V, Meinhardt T, Prüâ M, Reuter I, Schacherer F (2000). TRANSFAC: an integrated system for gene expression regulation. Nucleic Acids Res.

[B30] Matthews B (1975). Comparison of the predicted and observed secondary structure of T4 phage lysozyme. Biochim Biophys Acta.

[B31] Rissanen J (1986). Complexity of strings in the class of Markov sources. IEEE Trans Inform Theory.

[B32] Bühlmann P, Wyner AJ (1999). Variable length Markov chains. Ann Statist.

[B33] Reese MG, Eeckman FH, Kulp D, Haussler D (1997). Improved splice site detection in Genie. J Comput Biol.

[B34] Ketterling RP, Drost JB, Scaringe WA, Liao DZ, Liu JZ, Kasper CK, Sommer SS (1999). Reported in vivo splice-site mutations in the factor IX gene: severity of splicing defects and a hypothesis for predicting deleterious splice donor mutations. Hum Mutat.

[B35] Staley JP, Guthrie C (1999). An RNA switch at the 5' splice site requires ATP and the DEAD box protein Prp28p. Mol Cell.

[B36] Thanaraj T, Robinson AJ (2000). Prediction of exact boundaries of exons. Brief Bioinform.

[B37] Carmel I, Tal S, Vig I, Ast G (2004). Comparative analysis detects dependencies among the 5' splice-site positions. RNA.

[B38] Berkeley Drosophila Genome Project. http://www.fruitfly.org/seq_tools/datasets/Human/GENIE_96/splicesets.

[B39] BDGP: Splice Site Prediction by Neural Network. http://www.fruitfly.org/seq_tools/splice.html.

[B40] Christopher Burge Lab. http://genes.mit.edu/burgelab/maxent/ssdata.

[B41] Nelson K, Green M (1990). Mechanism for Cryptic Splice Site Activation During Pre-mRNA Splicing. PNAS.

[B42] Nandabalan K, Price L, Roeder GS (1993). Mutations in U1 snRNA bypass the requirement for a cell type-specific RNA splicing factor. Cell.

[B43] Salzberg S, Delcher A, Kasif S, White O (1998). Microbial gene identification using interpolated Markov models. Nucl Acids Res.

[B44] Ohler U, Harbeck S, Niemann H, Noth E, Reese M (1999). Interpolated markov chains for eukaryotic promoter recognition. Bioinformatics.

[B45] Crooks GE, Hon G, Chandonia JM, Brenner SE (2004). WebLogo: A Sequence Logo Generator. Genome Res.

