# Supplementary material for: Optimized mixed Markov models for motif identification
Source: BMC Bioinformatics. 2006 Jun 2;7:279. doi: 10.1186/1471-2105-7-279 (PMC1534070; doi:10.1186/1471-2105-7-279)
Supplement: Additional file 1 — The supplement includes the mathematical formulas for computing the probability of a motif site given a Markov model, the algorithmic pseudo-code for the DNJ method, and the description of the parameter estimation for our model. It also contains supplemental materials for the main results as well as other additional results, such as the application for protein domain identification, the comparison of computational time, and so on. [file 1471-2105-7-279-S1.pdf]

Supplementary Materials

Calculation of $\Pr(x|M_k^L)$ and $\Pr(x|M_k^C)$

In real applications, it is easier to calculate $\Pr(x|M_k^L)$ or $\Pr(x|M_k^C)$ in terms of probabilities of oligomers. In the following, we derived generalized formulas to calculate a motif probability given a Markov model. These generalized formulas also give us a clearer view of the differences in the probabilities of a motif sequence given different Markov models.

- **Linear chain models**

$$\Pr(x|M_0^L) = \Pr(x|M_0^C) = \prod_{i=1}^{w} \Pr(x_i) \tag{1}$$

$$\begin{aligned}\Pr(x|M_1^L) &= \Pr(x_1)\Pr(x_2|x_1)\Pr(x_3|x_2)\cdots\Pr(x_w|x_{w-1})\\ &= \frac{\Pr(x_1,x_2)\Pr(x_2,x_3)\cdots\Pr(x_{w-1},x_w)}{\Pr(x_2)\cdots\Pr(x_{w-1})}\\ &= \Pr(x_1,x_2)\prod_{i=2}^{w-1}\frac{\Pr(x_i,x_{i+1})}{\Pr(x_i)}\\ &= \prod_{i=1}^{w}\Pr(x_i)\times\prod_{i=1}^{w-1}\frac{\Pr(x_i,x_{i+1})}{\Pr(x_i)\Pr(x_{i+1})}\\ &= \Pr(x|M_0^L)\times\prod_{i=1}^{w-1}\frac{\Pr(x_i,x_{i+1})}{\Pr(x_i)\Pr(x_{i+1})}\end{aligned} \tag{2}$$

$$\begin{aligned}\Pr(x|M_2^L) &= \Pr(x_1,x_2)\Pr(x_3|x_1,x_2)\Pr(x_4|x_2,x_3)\cdots\Pr(x_w|x_{w-2},x_{w-1})\\ &= \frac{\Pr(x_1,x_2,x_3)\Pr(x_2,x_3,x_4)\cdots\Pr(x_{w-2},x_{w-1},x_w)}{\Pr(x_2,x_3)\cdots\Pr(x_{w-2},x_{w-1})}\\ &= \Pr(x_1,x_2,x_3)\prod_{i=2}^{w-2}\frac{\Pr(x_i,x_{i+1},x_{i+2})}{\Pr(x_i,x_{i+1})}\\ &= \prod_{i=1}^{w}\Pr(x_i)\times\prod_{i=1}^{w-1}\frac{\Pr(x_i,x_{i+1})}{\Pr(x_i)\Pr(x_{i+1})}\times\prod_{i=1}^{w-2}\frac{\Pr(x_{i+1})\Pr(x_i,x_{i+1},x_{i+2})}{\Pr(x_i,x_{i+1})\Pr(x_{i+1},x_{i+2})}\\ &= \Pr(x|M_1^L)\times\prod_{i=1}^{w-2}\frac{\Pr(x_{i+1})\Pr(x_i,x_{i+1},x_{i+2})}{\Pr(x_i,x_{i+1})\Pr(x_{i+1},x_{i+2})}\end{aligned} \tag{3}$$

$$\Pr(x|M_3^L) = \Pr(x|M_2^L)\times\prod_{i=1}^{w-3}\frac{\Pr(x_{i+1},x_{i+2})\Pr(x_i,x_{i+1},x_{i+2},x_{i+3})}{\Pr(x_i,x_{i+1},x_{i+2})\Pr(x_{i+1},x_{i+2},x_{i+3})} \tag{4}$$

$$\vdots$$

$$\Pr(x|M_k^L) = \Pr(x|M_{k-1}^L)\times\prod_{i=1}^{w-k}\frac{\Pr(x_{i+1},\ldots,x_{i+k-1})\Pr(x_i,\ldots,x_{i+k})}{\Pr(x_i,\ldots,x_{i+k-1})\Pr(x_{i+1},\ldots,x_{i+k})} \tag{5}$$

- **Circular chain models**

  Define $x_i = x_{i-w}$ if $i > w$

$$\begin{aligned}
\Pr(x|M_1^C) &= \Pr(x_2|x_1)\Pr(x_3|x_2)\cdots\Pr(x_w|x_{w-1})\Pr(x_1|x_w) \\
&= \frac{\Pr(x_1,x_2)\Pr(x_2,x_3)\cdots\Pr(x_{w-1},x_w)\Pr(x_1,x_w)}{\Pr(x_1)\Pr(x_2)\cdots\Pr(x_{w-1})\Pr(x_w)} \\
&= \frac{\Pr(x_1,x_w)}{\Pr(x_w)}\prod_{i=1}^{w-1}\frac{\Pr(x_i,x_{i+1})}{\Pr(x_i)} = \prod_{i=1}^{w}\frac{\Pr(x_i,x_{i+1})}{\Pr(x_i)} \\
&= \prod_{i=1}^{w}\Pr(x_i)\times\prod_{i=1}^{w}\frac{\Pr(x_i,x_{i+1})}{\Pr(x_i)\Pr(x_{i+1})} \\
&= \Pr(x|M_0^C)\times\prod_{i=1}^{w}\frac{\Pr(x_i,x_{i+1})}{\Pr(x_i)\Pr(x_{i+1})} = \Pr(x|M_0^C)\times\prod_{i=1}^{w}\frac{\Pr(x_i,x_{i+1})}{\Pr(x_i)^2}
\end{aligned} \tag{6}$$

$$\begin{aligned}
\Pr(x|M_2^C) &= \Pr(x_3|x_1,x_2)\cdots\Pr(x_w|x_{w-2},x_{w-1})\Pr(x_1|x_{w-1},x_w)\Pr(x_2|x_w,x_1) \\
&= \frac{\Pr(x_1,x_2,x_3)\cdots\Pr(x_{w-2},x_{w-1},x_w)\Pr(x_{w-1},x_w,x_1)\Pr(x_w,x_1,x_2)}{\Pr(x_1,x_2)\Pr(x_2,x_3)\cdots\Pr(x_{w-2},x_{w-1})\Pr(x_{w-1},x_w)\Pr(x_w,x_1)} \\
&= \prod_{i=1}^{w}\frac{\Pr(x_i,x_{i+1},x_{i+2})}{\Pr(x_i,x_{i+1})} \\
&= \prod_{i=1}^{w}\Pr(x_i)\times\prod_{i=1}^{w}\frac{\Pr(x_i,x_{i+1})}{\Pr(x_i)\Pr(x_{i+1})}\times\prod_{i=1}^{w}\frac{\Pr(x_{i+1})\Pr(x_i,x_{i+1},x_{i+2})}{\Pr(x_i,x_{i+1})\Pr(x_{i+1},x_{i+2})} \\
&= \Pr(x|M_1^C)\times\prod_{i=1}^{w}\frac{\Pr(x_{i+1})\Pr(x_i,x_{i+1},x_{i+2})}{\Pr(x_i,x_{i+1})^2}
\end{aligned} \tag{7}$$

$$\Pr(x|M_3^C) = \Pr(x|M_2^C)\times\prod_{i=1}^{w}\frac{\Pr(x_{i+1},x_{i+2})\Pr(x_i,x_{i+1},x_{i+2},x_{i+3})}{\Pr(x_i,x_{i+1},x_{i+2})^2} \tag{8}$$

$$\vdots$$

$$\Pr(x|M_k^C) = \Pr(x|M_k^C)\times\prod_{i=1}^{w}\frac{\Pr(x_{i+1},\ldots,x_{i+k-1})\Pr(x_i,\ldots,x_{i+k})}{\Pr(x_i,\ldots,x_{i+k-1})^2} \tag{9}$$

DNJ Algorithm

Algorithm 1: Algorithm for joining the two nearest nodes in the directed neighbor-joining method for k^{th} order Markov chain

`// Start with two nearest neighbors represented by vectors` $\overrightharpoon{u}$ `and` $\overrightharpoon{v}$`, respectively. The harpoon at top of` u `and` v `indicates the order of positions in` u `and` v`. For example, if` $\overrightharpoon{u} = (2,1,6,5)$ `and` $\overrightharpoon{v} = (7,3,4,8)$`, then` $\overleftharpoon{u} = (5,6,1,2)$`,` $\overleftharpoon{v} = (8,4,3,7)$`, and` $\overrightharpoon{u}\overleftharpoon{v} = (2,1,6,5,8,4,3,7)$`.` $\overrightharpoon{u}_i$ `denotes` i^{th} `position in vector` $\overrightharpoon{u}$`.`

Data: $\overrightharpoon{u}$ and $\overrightharpoon{v}$

M = number of positions in $\overrightharpoon{u}$;
N = number of positions in $\overrightharpoon{v}$;
if $M < N$ **then**
  Swap($\overrightharpoon{u}, \overrightharpoon{v}$);
  Swap(M, N) ;
endif
if $M == 1$ *and* $N == 1$ **then**
  `// Directly join` $\overrightharpoon{u}$ `and` $\overrightharpoon{v}$ `to form a new node`
  $\overrightharpoon{x} = \overrightharpoon{u}\overrightharpoon{v}$;
else if $N == 1$ *and* $M <= K$ **then**
  $m = \mathrm{int}(M/2)$;
  `// The distance of` $\overrightharpoon{v}$ `to left side of` $\overrightharpoon{u}$
  $L_{Left} = \sum_{i=1}^{m} d(\overrightharpoon{u}_i, \overrightharpoon{v}_1)$;
  `// The distance of` $\overrightharpoon{v}$ `to right side of` $\overrightharpoon{u}$
  $L_{Right} = \sum_{i=m+1}^{M} d(\overrightharpoon{u}_i, \overrightharpoon{v}_1)$;
  $\overrightharpoon{x} = \begin{cases} \overrightharpoon{v}\overrightharpoon{u} & \text{if } D_{Left} < D_{Right} \\ \overrightharpoon{u}\overrightharpoon{v} & \text{if } D_{Left} >= D_{Right} \end{cases}$;
else
  $m = \min(M, K)$ and $n = \min(N, K)$;
  $L_{\overleftharpoon{v},\overrightharpoon{u}} = \sum_{j=1}^{n} \sum_{i=j}^{m} d(\overrightharpoon{u}_i, \overrightharpoon{v}_j)$;
  $L_{\overrightharpoon{u},\overrightharpoon{v}} = \sum_{j=1}^{n} \sum_{i=j}^{m} d(\overrightharpoon{u}_{M-i+1}, \overrightharpoon{v}_j)$;
  $L_{\overrightharpoon{v},\overrightharpoon{u}} = \sum_{j=1}^{n} \sum_{i=j}^{m} d(\overrightharpoon{u}_i, \overrightharpoon{v}_{N-j+1})$;
  $L_{\overrightharpoon{u},\overleftharpoon{v}} = \sum_{j=1}^{n} \sum_{i=j}^{m} d(\overrightharpoon{u}_{M-i+1}, \overrightharpoon{v}_{N-j+1})$;
  $L_{min} = \min\left(L_{\overleftharpoon{v},\overrightharpoon{u}}, L_{\overrightharpoon{u},\overrightharpoon{v}}, L_{\overrightharpoon{v},\overrightharpoon{u}}, L_{\overrightharpoon{u},\overleftharpoon{v}}\right)$;
  $\overrightharpoon{x} = \begin{cases} \overleftharpoon{v}\overrightharpoon{u} & \text{if } L_{\overleftharpoon{v},\overrightharpoon{u}} = L_{min} \\ \overrightharpoon{u}\overrightharpoon{v} & \text{if } L_{\overrightharpoon{u},\overrightharpoon{v}} = L_{min} \\ \overrightharpoon{v}\overrightharpoon{u} & \text{if } L_{\overrightharpoon{v},\overrightharpoon{u}} = L_{min} \\ \overrightharpoon{u}\overleftharpoon{v} & \text{if } L_{\overrightharpoon{u},\overleftharpoon{v}} = L_{min} \end{cases}$;
endif
return $\overrightharpoon{x}$

Estimation of model parameters

For the kth order Markov model, the length of all possible oligomers ranges from 1 to $k+1$. We can estimate the probabilities of all these oligomers, as model parameters, by fitting the model with training data. Let $Y = Y_1, \ldots, Y_v$ be a random vector associated with the oligomers consisting of bases from some certain positions of a motif, then Y follows a multinomial distribution:

$$\Pr(Y|\theta) = \binom{N}{y_1, \ldots, y_v} \prod_{i=1}^{v} \theta_i^{y_i} \tag{10}$$

where N is the total number of motif sequences, v is the number of all possible y_i, y_i is the number of oligomer i and $\sum_{i=1}^{v} y_i = N$, and θ_i is the probability of oligomer i occurred and $\sum_{i=1}^{v} \theta_i = 1$. According to the Bayes' theorem, the probability of θ given Y can be expressed as

$$\Pr(\theta|Y) = \frac{\Pr(Y|\theta) \cdot \Pr(\theta)}{\Pr(Y)} \propto \Pr(Y|\theta) \cdot \Pr(\theta) \tag{11}$$

Suppose that the prior distribution of θ is a Dirichlet distribution as given by equation (12), then we can estimate the posterior mean of θ by maximum likelihood (equations 13 and 14).

$$\Pr(\theta_i|\alpha) = \frac{\Gamma(\sum_{i=1}^{v} \alpha_i)}{\prod_{i=1}^{v} \Gamma(\alpha_i)} \prod_{i=1}^{v} \theta_i^{\alpha_i - 1} \tag{12}$$

$$\hat{\theta} = \text{argmax}_{\theta} \log \left(Pr(Y|\theta) \Pr(\theta|\alpha) \right) \tag{13}$$

$$\hat{\theta}_i = \frac{y_i + \alpha_i - 1}{N + \sum_{i=1}^{v} \alpha_i - v} \tag{14}$$

where α is the parameter of Dirichlet distribution. In the context of Markov motif models, α is equivalent to pseudo counts of oligomers. For all our main results, we set pseudo counts = $\max(4, 0.01N)$, where N is the number of true motif sites in training dataset. The prior was chosen based on a rule of thumb without being optimized for performance.

Detection of protein domains

OMiMa is a versatile motif prediction tool for biological sequences, including DNA, RNA and protein sequences. In this evaluation, we tested OMiMa's capability for motif identification in protein sequences. The functional motifs in protein sequences are also known as function domains that are usually well-conserved across different members in the same protein family. The data for this evaluation are the alignment seeds of Pfam protein domains (`ftp://ftp.sanger.ac.uk/pub/databases/Pfam/Pfam-A.seed.gz`). We used a domain named *"A Propetide"* (accession # PF07966), of which the length is 29 bases. In total, there are 86 true domain sites, and for each true domain site, 50 false domain sites are simulated by randomly shuffling the amino acids of the true site. For the 0-1 mixture model, OMiMa optimized position arrangement for the first order chain is given by

`5-3-15-7-10-12-26-9-28-13-1-24-21-16-20-0-2-14-11-6-25-17-27-8-22-18-23-4-19`

Although no position is independent of all others for this domain motif (the length of zero order chain is 0), the best model chosen by OMiMa (AIC criterion) is the zero order Markov model. We compared the following three Markov models: zero order, un-optimized first order, and DNJ optimized first order models by 10-fold cross validation. The log-likelihood ratio was used to score a domain site, and the first order Markov chains were set to linear. Results (not given here) show that both the zero order, and DNJ optimized Markov models have perfect prediction (both Sn and Sp are 1), while the un-optimized first order Markov model does not.

Computational efficiency

OMiMa is an efficient computational tool designed for finding motifs in large genome sequences. Using Reese's dataset of human donor sites, we compared the running times used by OMiMa, PVLMM, and NNSplice. Since PVLMM and OMiMa run on different computer operating systems, the test was performed on a SUSE Linux/Window XP dual-boot computer with Intel 3.0 GHZ CPU and 512 M main memory. NNSplice is a web-based program at `http://www.fruitfly.org/seq_tools/splice.html`, so we calculated its computational time as the interval from submitting data to returning the result from the NNSplice web-server using Microsoft IE browser on the same dual-boot computer. Results (Table 1) showed that OMiMa is much more efficient than either NNSplice or PVLMM.

Tables and Figures

Table 1: Computational time used by OMiMa, NNSplice and PVLMM for donor site prediction. OMiMa's 1-L-1/1-C-1 model and the first order PVLMM (with depth 1) were used for this comparison. The running times of OMiMa and PVLMM include times for both model training and motif finding in testing data.

Tools	OMiMa	PVLMM	NNSplice
Model training time	$< 1\ s$	$34\ min\ 45\ s$	NA
Motif searching time	$< 1\ s$	$9\ s$	$25\ s$

Table 2: Comparison of OMiMa, PVLMM and MEM for donor site prediction by three-fold cross-validation. The table shows the number of true or decoy sites in each data partition for the cross-validation.

	Training		Testing	
Partition	true sites	decoy sites	true sites	decoy sites
0	8416	179437	4207	89718
1	8415	179436	4208	89719
2	8415	179437	4208	89718

Table 3: Comparison of donor site prediction by OMiMa: Models trained by the full Yeo's original training dataset *vs* Models trained by only 60% data from the orignial one. Compared to MEM, our OMiMa requires fewer training samples for the models with similar performance. We showed here that OMiMa was able to retain similar performance even when trained by only 60% data from the original traning set. In this assessment, we obtained 5 new training sets, each of which has only 60% data from the Yeo's original training dataset. The motifs in the each new training set were randomly sampled (without replacement) from the original training dataset. The testing dataset is the same Yeo's original testing dataset for all trained models. For models trained by 60% data from the orignial one, the results shown here are averaged values over the 5 new training sets (the detailed results of each training set are available at OMiMa online supplementary materials).

	Ac Maximized			Mc Maximized		
Model	Sn	Sp	Ac	Sn	Sp	Mc
	full original training set					
2-L-1	0.534	0.989	0.968	0.810	0.968	0.643
2-C-1	0.524	0.989	0.968	0.782	0.972	0.647
3-L-1	0.582	0.987	0.969	0.798	0.971	0.654
3-C-1	0.531	0.989	0.969	0.774	0.975	0.658
	60% of the original training set					
2-L-1	0.536	0.989	0.968	0.778	0.972	0.645
2-C-1	0.572	0.987	0.968	0.793	0.971	0.650
3-L-1	0.569	0.988	0.969	0.791	0.972	0.653
3-C-1	0.609	0.986	0.969	0.784	0.973	0.655

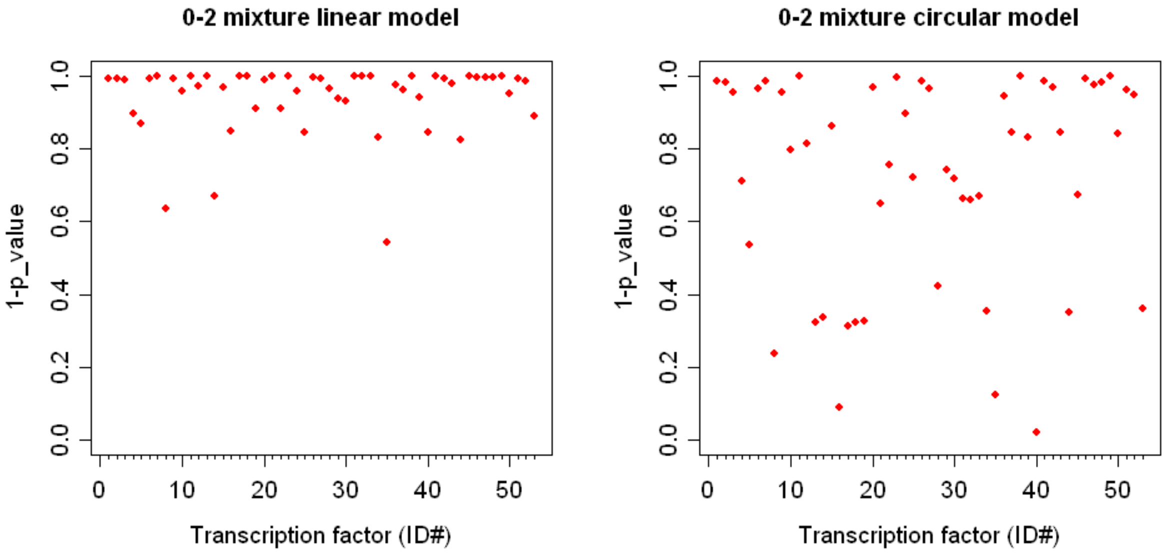

(a) Linear chain (b) Circular chain

Figure 1: Performance of DNJ optimized 0-2 mixture models of TFBS. The values on x-axis are the ID numbers of 53 TFBS. The y-axis is 1-p_value measuring performance of the DNJ optimized models relative to the randomly permuted models. a) 0-2 mixture linear models, b) 0-2 mixture circular models.

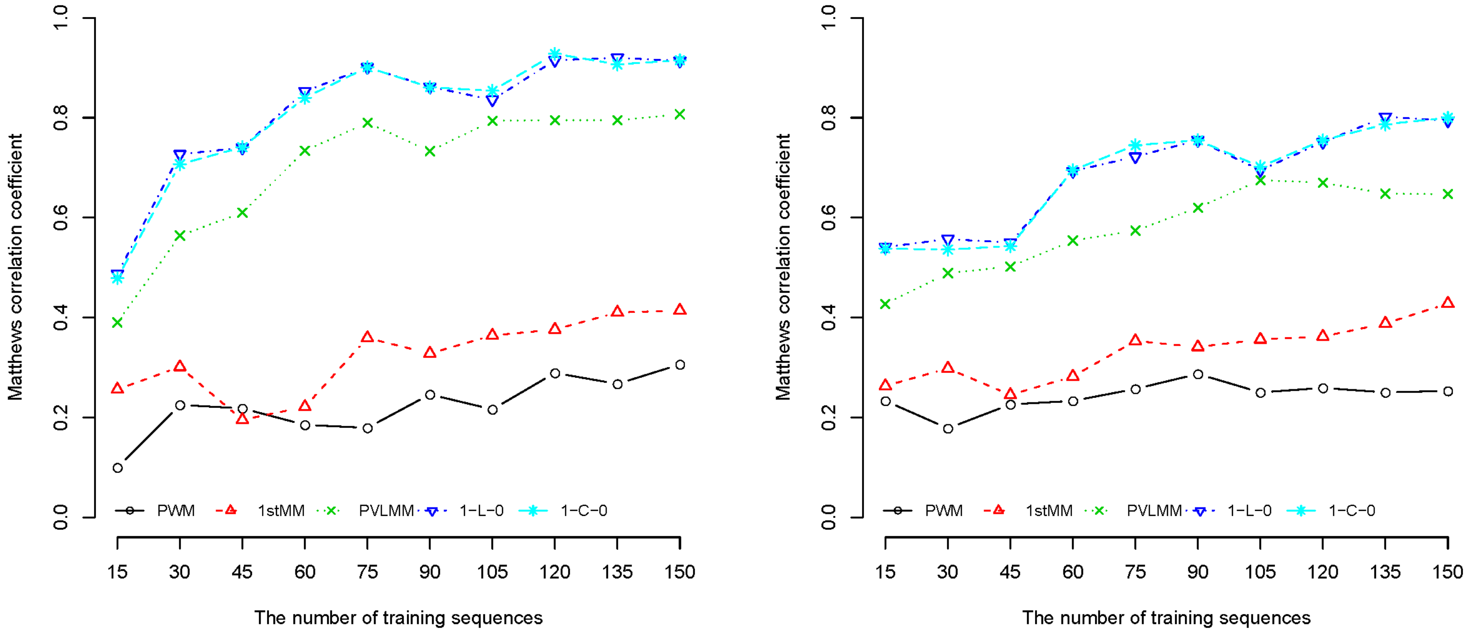

(a) TFBS A

(b) TFBS B

Figure 2: The performance comparison of different methods for predicting the simulated palindromic TFBS A and B. The x-axis shows the number of motif sequences used for training. The y-axis is the Matthews correlation coefficient of each method in predicting the same testing dataset (150 false and 150 true sites, respectively). The figure shows that OMiMa (1-L-0 and 1-C-0) performed significantly better than the other methods, regardless the size of training set. a) TFBS A, b) TFBS B.

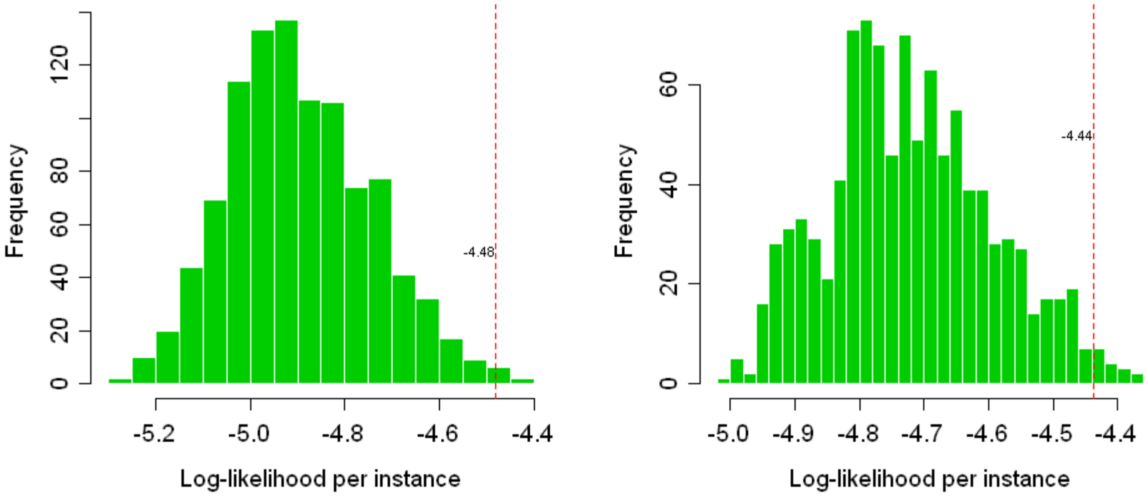

(a) Linear chain (b) Circular chain

Figure 3: Modeling V$AP1_Q4_01 TFBS The histogram is the log-likelihood score distribution of 1,000 randomly permuted mixture model. The red reference line indicates the log-likelihood score of the DNJ optimized model. a) 0-2 mixture linear model, b) 0-2 mixture circular model.

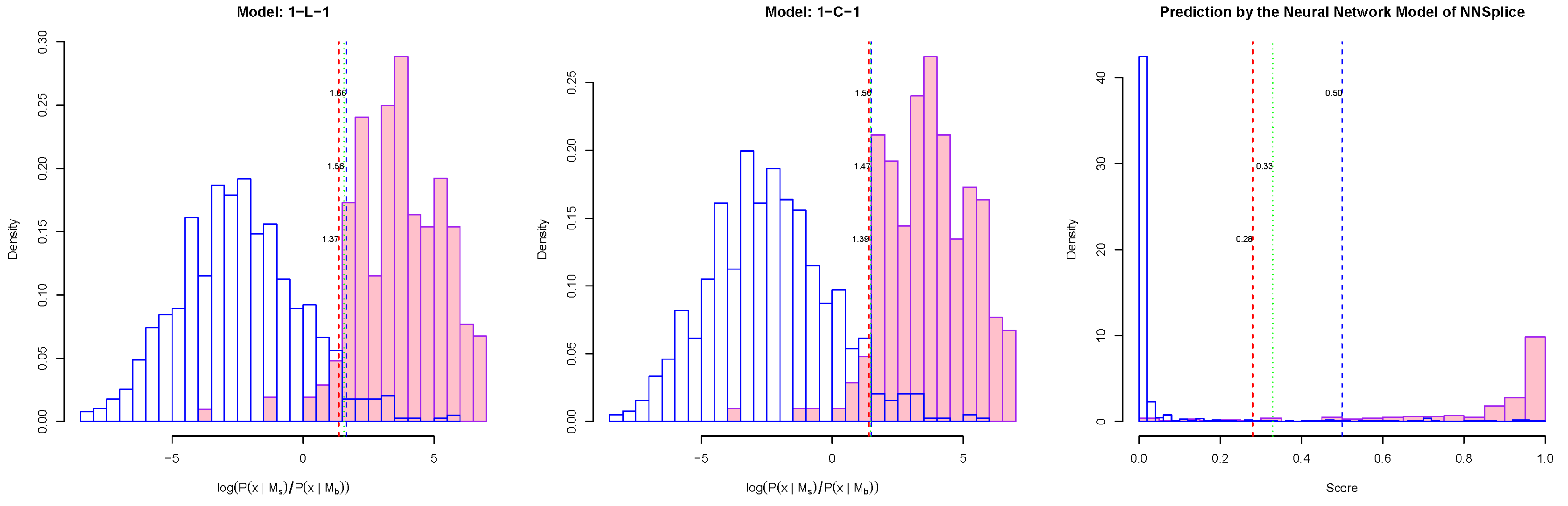

(a) Mixture model 1-L-1 (b) Mixture model 1-C-1 (c) NNSplice's neural network model

Figure 4: Comparing score distributions of different models for donor site prediction The score distributions of false (blue) and true (pink) sites. A site is predicted as a positive site if its score is larger than a defined cutoff. Three reference lines show 3 different cutoffs chosen by one of the following 3 criteria: balanced Sn and Sp (red dashed line), maximum of Mc (green dotted line), and maximum of Ac (blue dashed line). **(a)** 1-L-1, **(b)** 1-C-1, **(c)** NNSplice's neural network model.

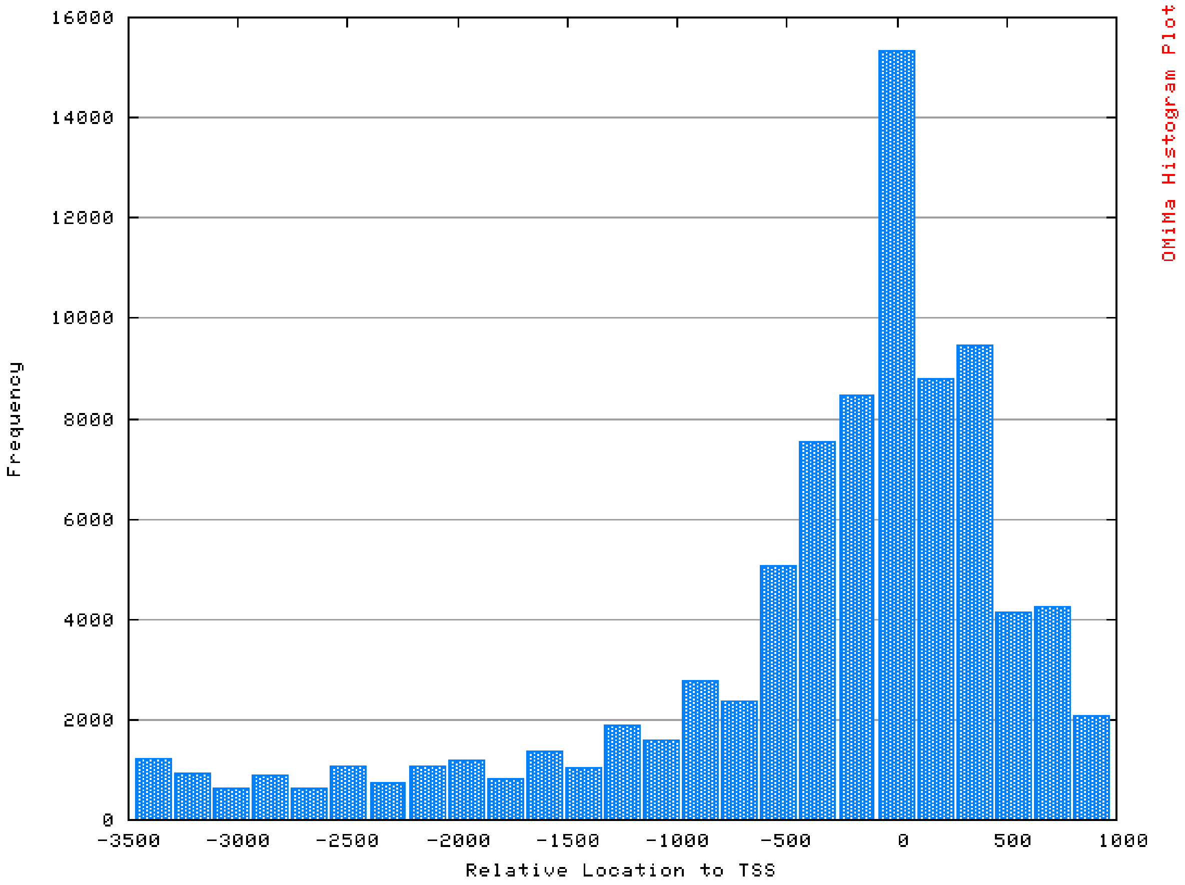

Figure 5: An example of histogram plot created by OMiMa. The plot shows the distribution of E2F binding sites relative to Transcription Start Sites (TSS) in the human genome.
